# Is transcranial direct current stimulation, alone or in combination with antidepressant medications or psychotherapies, effective in treating major depressive disorder? A systematic review and meta-analysis

**DOI:** 10.1186/s12916-021-02181-4

**Published:** 2021-12-17

**Authors:** Jingying Wang, Huichun Luo, Rasmus Schülke, Xinyi Geng, Barbara J. Sahakian, Shouyan Wang

**Affiliations:** 1 Institute of Science and Technology for Brain-Inspired Intelligence, Fudan University, 220 Handan Road, Yangpu District, Shanghai, 200433 China; 2 Key Laboratory of Computational Neuroscience and Brain-Inspired Intelligence (Fudan University), Ministry of Education, Shanghai, China; 3 Shanghai Engineering Research Center of AI & Robotics, Fudan University, Shanghai, China; 4 Engineering Research Center of AI & Robotics, Ministry of Education, Fudan University, Shanghai, China; 5 Shanghai Key Laboratory of Psychotic Disorders, Shanghai Mental Health Center, Shanghai Jiao Tong University School of Medicine, Shanghai, China; 6 Department of Psychiatry, Social Psychiatry and Psychotherapy, Hannover Medical School, Hannover, Germany; 7 Department of Psychiatry, University of Cambridge, Cambridge, UK; 8 Behavioural Clinical Neuroscience Institute, University of Cambridge, Cambridge, UK

**Keywords:** Transcranial direct current stimulation, Major depressive disorder, Treatment strategy, Combination therapy, Antidepressant, Systematic review, Meta-analysis

## Abstract

**Background:**

Transcranial direct current stimulation (tDCS) has shown mixed results for depression treatment. The efficacies of tDCS combination therapies have not been investigated deliberately. This review aims to evaluate the clinical efficacy of tDCS as a monotherapy and in combination with medication, psychotherapy, and ECT for treating adult patients with major depressive disorder (MDD) and identified the factors influencing treatment outcome measures (i.e. depression score, dropout, response, and remission rates).

**Methods:**

The systematic review was performed in PubMed/Medline, EMBASE, PsycINFO, Web of Sciences, and OpenGrey. Two authors performed independent literature screening and data extraction. The primary outcomes were the standardized mean difference (SMD) for continuous depression scores after treatment and odds ratio (OR) dropout rate; secondary outcomes included ORs for response and remission rates. Random effects models with 95% confidence intervals were employed in all outcomes. The overall effect of tDCS was investigated by meta-analysis. Sources of heterogeneity were explored via subgroup analyses, meta-regression, sensitivity analyses, and assessment of publication bias.

**Results:**

Twelve randomised, sham-controlled trials (active group: *N* = 251, sham group: *N* = 204) were included*.* Overall*,* the integrated depression score of the active group after treatment was significantly lower than that of the sham group (*g* = − 0.442, *p* = 0.017), and further analysis showed that only tDCS + medication achieved a significant lower score (*g* = − 0.855, *p* < 0.001). Moreover, this combination achieved a significantly higher response rate than sham intervention (OR = 2.7, *p* = 0.006), while the response rate remained unchanged for the other three therapies. Dropout and remission rates were similar in the active and sham groups for each therapy and also for the overall intervention. The meta-regression results showed that current intensity is the only predictor for the response rate. None of publication bias was identified.

**Conclusion:**

The effect size of tDCS treatment was obviously larger in depression score compared with sham stimulation. The tDCS combined selective serotonin re-uptake inhibitors is the optimized therapy that is effective on depression score and response rate. tDCS monotherapy and combined psychotherapy have no significant effects. The most important parameter for optimization in future trials is treatment strategy.

**Supplementary Information:**

The online version contains supplementary material available at 10.1186/s12916-021-02181-4.

## Background

Major depressive disorder (MDD) is a common illness worldwide, with more than 264 million people affected [1, 2]. Although there are effective pharmacological and physical treatments for MDD, about 50% of patients show an inadequate response to an individual antidepressant trial [3], about 25.5% show no response to electroconvulsive therapy [4], and 15.5–69% show insufficient response to repetitive transcranial magnetic stimulation [5]. Moreover, about 10% of patients become chronic (i.e. 2 years without clinical remission) and experience severe and cognitive impairment as well as psychosocial disability [6]. Therefore, there is clearly a need to consider other possible treatments for MDD and to consider what combined treatment might increase the efficacy of pharmacological treatment.

The number of clinical trials investigating the efficacy of transcranial direct current stimulation (tDCS) is rapidly increasing [7] due to its non-invasive nature, few side effects [8], and low economic burden [9]. This exploratory treatment has been tested widely for the treatment of major depressive disorder (MDD) [10–13]. Both clinical trials and systematic reviews with meta-analyses have shown that tDCS not only has the potential to improve mood symptoms in depressive patients [11, 14, 15] but is also able to enhance cognitive functions [16–18]. TDCS showed potential in patients with bipolar disorder in a major depressive episode [19–21] and postpartum depression [22], as well as in adult [23, 24] and elderly [25] populations.

Recent systematic reviews on the efficacy of tDCS on MDD have shown that it could result in small to moderate improvement in depression scores (Hedge’s *g* = 0.3–0.76) [15, 20, 26], a non-significant dropout rate (4.8–14.7%) [20, 26–28], and slightly higher response rate (23.3–34%) and remission rate (12.2–23.1%) [15, 20, 27–29], compared with sham stimulation. However, tDCS has shown inconsistent and uncertain outcomes in depression treatment [30]. A large multi-centre randomized controlled trial (RCT), Brunoni’s SELECT study [31], showed that the outcome of tDCS treatment was superior to that of sham treatment in a study of 120 MDD patients. In contrast, Loo et al.’s multi-centre RCT [32] indicated negative results: no difference in antidepressant effects was found between active and sham stimulation in a study of 84 patients with MDD.

These inconsistent findings might be caused by the differences in study design and variability of tDCS parameters among different RCTs [33, 34]. In reviewing previous studies, we found that factors such as demographics (e.g. age range, sex ratio) [34, 35], clinical characteristics (e.g. depression subtype, the severity of depression) [28, 35], and montages (e.g. current intensity, stimulation duration, and number of sessions) [28, 33] have been investigated as factors influencing the efficacy of tDCS. However, how these factors and their interactions influence tDCS effects remains an open question.

Besides the influencing factors mentioned above, tDCS is often used in addition to other treatments in clinical practice, such as medication [36], psychotherapy [37, 38], or electroconvulsive therapy (ECT) [39]. Such combination therapies could be either an influencing factor to decrease the treatment efficacy [15, 26, 27] or a potential approach to improve the efficacy of treatment for major depression [25, 28, 33]. However, so far, treatment strategies combining tDCS with other therapeutic approaches have not been getting enough attention.

In this systematic review, we asked whether tDCS is an effective treatment of MDD. We also wanted to determine whether tDCS could increase the efficacy of pharmacological or psychological treatments and which factors affect the efficacy of tDCS treatment. Therefore, we studied the outcomes of combination therapies including tDCS for major depression, by performing a meta-analysis of randomized, sham-controlled trials of adult patients with unipolar depression, to investigate the influence of treatment strategy on depression score, dropout rate, response rate, and remission rate. We aimed to (1) examine tDCS with and without combination therapies among four types of treatment strategies (i.e. tDCS alone, tDCS combines with medication, tDCS combines with psychotherapy, and tDCS combines with ECT), (2) identify the risk and protective factors from clinical characteristics, demographics, and montage parameters for treatment outcomes, and (3) verify the robustness of the conclusion by using sensitivity analysis, risk of bias assessment, and publication bias evaluation.

## Method

The study was registered on the International Prospective Register of Systematic Reviews, PROSPERO (CRD42020148953).

### Study selection

#### Search strategy and eligibility criteria

The study followed the PRISMA guidelines [40]. A systematic literature review was conducted using PubMed, Embase (accessed via Ovid), PsycINFO, ISI: Web of Knowledge, and OpenGrey databases. The following search terms were employed:

(“depress*” OR “major depressive disorder” OR “MDD”) AND (“transcranial direct current stimulation” OR “tDCS”)

This study followed strict inclusion criteria to ensure that the findings would be more conclusive. Studies were included based on the following criteria: (i) adult participants aged > 18 years with a diagnosis of MDD; (ii) studies that examined tDCS as a treatment for depression; (iii) randomised, sham-controlled trials, where the sham was either sham of tDCS or sham of tDCS + other therapies; (iv) stimulation targeting the dorsolateral prefrontal cortex; (v) tDCS protocols with at least five tDCS stimulation sessions on consecutive days; and (vi) inclusion of a clinician-administered depression rating scale, i.e. the Hamilton Depression Rating Scale (HDRS) [41] of any version or the Montgomery–Åsberg Depression Rating Scale (MADRS) [42] at baseline and post-treatment; and (vii) articles written in English. Studies that were excluded were as follows: (i) animal studies; (ii) those in which the participants were elderly, minors, or pregnant women; (iii) those investigating other types of depression, such as seasonal affective disorder; (iv) those involving comorbidity of depression with other neuropsychiatric diseases, such as bipolar disorder, personality disorders, Alzheimer’s disease, stroke, etc.; (v) studies lacking a tDCS sham-control group; and (vi) studies lacking any of the primary outcomes (i.e. depression score or dropout rate).

#### Screening procedure

**Fig. 1:**
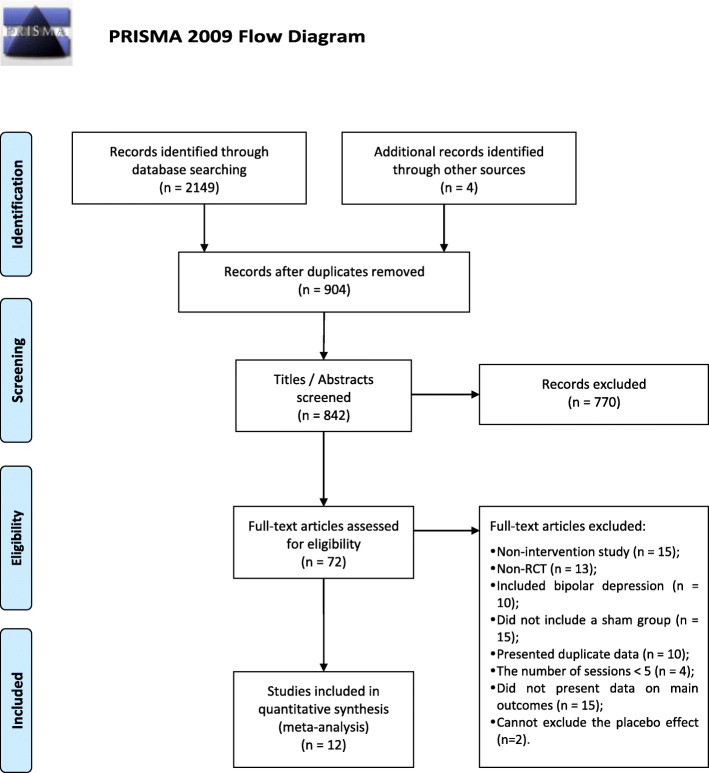
PRISMA study selection flowchart. The sum of excluded studies for each reason is not equal to the total number because some articles have more than one exclusion reason

The screening procedures were independently conducted by two authors (JW and HL). We searched the databases from January 1, 2006 (the first year available), until August 17, 2020. The screening procedure involved four steps, as shown in Fig. 1. Initially, duplicated publications were removed after identifying records from the five databases. Second, titles and abstracts were assessed for relevancy and reviewed to confirm whether the study met the inclusion and exclusion criteria. Third, the full text was read to confirm if the study still met the exclusion criteria when it could not be confirmed in the second step (excluded publications are listed in Additional file 1: S1). Finally, the two resulting publication lists were compared. They disagreed on two studies [23, 24], which were then excluded because of the lack of a sham group and inclusion of inseparable bipolar patients. The final list of the selected publications is presented in Additional file 1: S2.

### Data extraction

We extracted information on literature (authors, year of publication), clinical characteristics, stimulation parameters, and intervention outcomes. Clinical characteristics included sample size, age (mean, standard deviation), sex, depression severity scores (mean, standard deviation) at baseline, treatment strategy (monotherapy or combination therapy), and allocation to either sham or active tDCS. Combination therapy was called augmentation or add-on treatment, according to whether the timing of therapies is concurrent or not [26]. In this review, the subtypes of treatment strategy we distinguished without considering simultaneousness, but considering which therapy or therapies were applied, are shown in Table 1.

**Table 1:** Subtypes of treatment strategy

Treatment strategy	
*Monotherapy*	
	tDCS alone
*Combination therapy*	
	tDCS + medication
	tDCS + psychotherapy
	tDCS + ECT

Stimulation parameters extracted included the number of electrodes, electrode size, current intensity, stimulation duration, number of sessions, and total charge (defined as the amount of charge received by the subject during all tDCS sessions, which equals the current intensity × stimulation duration × number of sessions/size of electrode) [43].

Intervention outcomes extracted included the depression severity score (mean and standard deviation), dropout rate, response rate, and remission rate at the immediate post-treatment endpoint.

All relevant information was extracted from eligible papers by two authors (JW and HL) independently and were then inspected by one author (JW). Discrepancies were discussed by the two investigators. If data could not be accessed from the original publications, it was requested from the relevant corresponding author, or extracted from other reviews or articles.

### Outcome measures

The measures of primary outcomes were severity (i.e. depression score) measured by the HDRS or MADRS after treatment (i.e. the score at the immediate post-treatment endpoint) and acceptability of tDCS (i.e. dropout rate, which is the rate of participants who dropped out in the active or sham group at the endpoint). The secondary outcomes were response rate and remission rate. Response was defined as a 50% or greater reduction in the depression score from the baseline. Remission was defined according to the criteria used in each trial (e.g. HDRS-17 score ≤ 7 or MADRS score ≤ 10). If studies reported scores on both the HDRS and MADRS, the scores we selected for analyses were from the scale utilised for defining the response and remission in their trials.

### Data analysis

The Cochrane tool [44] was used to evaluate the risk of bias in included RCTs. This instrument of risk of bias was used to assess two types of selection bias, performance bias, detection bias, attrition bias, and reporting bias, according to standardised criteria. Each trial received a score of low, high, or unclear risk of bias for each type of bias. Additionally, the overall risk of bias was evaluated according to the above six types of bias, according to Mutz et al. [45]. Low overall risk of bias is defined as low risk in all domains or unclear risk in one domain. Unclear overall risk of bias is regarded as at least two domains with an unclear risk of bias. High overall risk of bias means at least one domain with a high risk. Two raters (JW and HL) independently assessed the risk of bias. The graphs summarizing and showing the distribution of risk were produced with RevMan (version 5.3, Cochrane Organization, London, UK).

Statistical analyses were performed using the Stata software (Version 16.0, Stata Corp., College Station, TX, USA). We used a random-effects pooling model for all analyses. We pooled the effect sizes using the DerSimonian and Laird (D + L) method, with the Hartung–Knapp adjustment for the random-effects model. Analyses were performed in intention-to-treat samples. Moreover, we only analysed immediate after-effects, which are the immediate post-treatment endpoint outcomes, rather than the outcomes at follow-up visits. For studies that presented zero events in response/remission/dropout outcomes in any group, we used a continuity correction of 0.5 [46]. However, if the events in the two groups were zero, this trial would be excluded automatically by Stata.

Baseline variables were analysed to compare differences between the active and sham groups. Paired-sample *t* tests or Mann-Whitney *U* tests were used for paired or independent two-group variables, respectively.

Meta-analyses of all outcomes were performed using random-effects models with 95% confidence intervals (95% CI). Outcomes were assessed with both continuous measures of depression scores and categorical measures of response rate, remission rate, and dropout rate. The effect sizes of measures were evaluated with Hedge’s *g* for continuous outcome measures and odds ratios (ORs) for categorised outcomes. Hedges’ *g* of 0.2 is considered small, 0.5 as moderate, and 0.8 as large [47]. OR > 1 is regarded as indicative of favouring active treatment (positive); otherwise, OR < 1 indicates favouring sham treatment (negative). Effect sizes were computed according to the random-effects model. Heterogeneity was evaluated using the *χ*^2^-based *Q* test and metric *I*^2^; *p* < 0.1 for the *Q* test indicates significant heterogeneity and *I*^2^ > 25%, 50%, and 75% indicates a low, moderate, and high heterogeneity, respectively [48].

Subgroup analyses were performed to further compare the differences in the primary and secondary outcomes between the active and sham groups for each treatment strategy; effect sizes were calculated according to the random-effects model. To explore the influence of clinical characteristics (i.e. publication year, baseline score, and treatment strategy), demographics (i.e. sample size, age, and female proportion), and montage parameters (i.e. electrode size, current intensity, stimulation duration, number of sessions, and total charge) on outcome measures (i.e. depression score, dropout rate, response rate, and remission rate), univariate meta-regression models with dummy variables (i.e. treatment strategy) were performed. If more than one variable had a *p* value < 0.1, stepwise regression (backward method) was conducted by inputting these variables to analyse each variable’s contribution further [28]. Finally, factors were considered significant based on *p* < 0.05.

Sensitivity analyses, including influence analysis and meta-analysis without trials with a high-risk bias, were conducted to assess the robustness of the primary and secondary outcome measures. Influence analysis qualitatively evaluated the influence of each study on the net results by sequentially excluding one study at a time. In addition, a meta-analysis was implemented to analyse the robustness quantitatively, by excluding trials with a high overall risk of bias. Publication bias was quantitatively evaluated using Egger's regression intercept test. Funnel plots were visually evaluated for the presence of small study effects.

## Results

### Overview

Twelve randomised controlled trials, consisting of 29 treatment arms, met the inclusion criteria. For the study of Brunoni et al. [31], two of the study arms (active tDCS + medication and sham tDCS + medication) were retained and two (tDCS + placebo and sham + placebo) arms were discarded, as placebo-effect was not considered in this study. The active group with a target located in the occipital cortex was excluded in the study by Boggio et al. [49] since only studies targeting the dorsal lateral prefrontal cortex (dlPFC) were considered in this meta-analysis. In Segrave et al.’s trial [50], the tDCS + sham cognitive control training group was not included in further analysis. Pavlova et al.’s study [51] had three arms, including one sham and two active arms; the sample size of the sham group was split into halves, as it was calculated twice in the paper.

Overall, 25 treatment arms were included. A total of 455 patients (mean age = 45.7 years, 58.2% female) of whom *n* = 251 were randomised to active and *n* = 204 to sham treatments. There was no significant difference in the number of participants (*t*_12_ = 1.2, *p* = 0.255) and age (mean: *t*_12_ = 0.477, *p* = 0.642; SD: *t*_12_ = 0.223, *p* = 0.827) between the two groups, but significantly more males (*t*_12_ = 2.61, *p* = 0.023) were included in the active group. The baseline depression scores were 24.36 ± 5.74 (mean ± SD) and 25.36 ± 5.13 (mean ± SD) for the active and sham groups, respectively. Paired-samples *t* test revealed no significant difference in the baseline depression score (mean: *t*_12_ = − 1.08, *p* = 0.301; SD: *t*_12_ = 1.84, *p* = 0.091) between the two groups.

Studies generally had small sample sizes (mean = 37.9, SD = 22.4). All studies applied unilateral or bilateral dlPFC stimulation, with anodal stimulation applied over the left dlPFC, and cathodal stimulation applied over the right dlPFC or other places. Most studies used setups involving two electrodes (91.7%), 1 (16.7%) or 2 mA electric currents (66.7%), 20- (41.7%) or 30-min duration (58.3%), and 10 (50%) or > 10 (25%) sessions (Table 2).

**Table 2:** Study characteristics for each trial

First author (year)	Treatment strategy	Location		Electrode size (cm^2^)	Number of electrodes	Current (mA)	Session duration (min)	Session number (*n*)	Total charge	*N*	Age (mean)	Female ratio	Baseline score	
		Anode	Cathode/reference										Active	Sham
Boggio (2008) ^1^ [49]	mono	F3	RSA	35	2	2	20	10	11.4	31	49.4	0.70	21.1 ± 4.4	21.9 ± 4.8
Loo (2010)^1^ [12]	mono	F3	F8^a^	35	2	1	20	5	2.9	40	45.6	0.55	18.3 ± 5.75	17.25 ± 4.7
Blumberger (2012)^1^ [52]	mono	F3	F4	35	2	2	20	15	17.1	24	47.3	0.83	24.9 ± 3.1	24.1 ± 2.9
Loo (2018)^2^ [32]	mono	F3	F8^b^	35	2	2.5	30	20	17.1	84	46.7	0.50	29.05 ± 5.01	27.69 ± 5.56
Bennabi (2015) ^1^ [36]	+med	F3	FP2	35	2	2	30	10	42.9	23	61.8	0.65	22.7 ± 5.3	24.2 ± 5.6
Brunoni (2013) ^2^ [31]	+med	F3	F4	25	2	2	30	10	24	60	41.0	0.68	30.73 ± 6.72	30.50 ± 6.81
Pavlova (2018) ^1^ [51]	+med	F3	F8^a^	17.5	2	0.5	20 30	10	5.7 8.6	69	37.6	0.63	17.8 ± 3.7^3^ 16.3 ± 3.1^4^	16.6 ± 2.5
Brunoni (2014) ^1^ [16]	+psych*	F3	F4	25	2	2	30	10	24	37	43.8	0.30	25.6 ± 5.8	27 ± 5.7
Segrave (2014) ^2^ [50]	+psych*	F3	F8^a^	35	2	2	24	5	6.9	18	40.4	0.33	27 ± 5.57	27.11 ± 4.68
Welch (2018) ^1^ [37]	+psych^#^	F3	F4	25	2	2	30	12	28.8	14	53.2	0.86	20.9 ± 13.7	32.2 ± 11.44
Nord (2019) ^1^ [38]	+psych^#^	F3	ID	35	1	1	20	8	4.6	39	35.6	0.51	21.95 ± 3.20	21.05 ± 3.27
Mayur (2018) ^2^ [39]	+ECT	F3	F8^b^	25	2	2	30	10	24	16	45.0	0.38	29.5 ± 10.16	32.38 ± 6.44

^1^Baseline score was assessed by HDRS

^2^Baseline score was assessed by MADRS

^3^Duration of stimulation = 20 min

^4^Duration of stimulation = 30 min

*Treatment strategy*: mono, tDCS monotherapy; +med, tDCS +medication therapy; +psych, tDCS +psychotherapy therapy (*cognitive control therapy; ^#^cognitive behavioural therapy); +ECT, tDCS +ECT therapy

*Location*: F3- left DLPFC, F4- right DLPFC, F8-lateral contralateral orbit(a)/lateral right frontal(b)/, FP2-contralateral supraorbital area, RSA: right supraorbital area, ID: ipsilateral deltoid. Electrode locations were reported according to the International 10/20 system

Total charge defined as the amount of charge received by the subject during all tDCS sessions, which equals (current intensity × stimulation duration × number of sessions)/electrode size [43]

*N* represents sample size that applied to data analysis

### Risk of bias assessment

The risk of bias assessment showed that there were five studies with low risk, five with unclear risk, and two with high risk among the 12 trials in terms of the overall bias, according to the Cochrane risk of bias analysis (Additional file 2: S1(a)). Two studies were at high risk of bias because of a lack of the outcomes response and remission rates and ineffective blinding for the active group, respectively (Additional file 2: S3). The quality assessment indicated that the studies presented a low risk concerning attrition bias (91.7%) and reporting bias (91.7%). However, about half of the trials showed low risk concerning randomization bias (58.3%), allocation concealment bias (58.3%), blinding of participant bias (58.3%), and blinding of raters’ bias (75%) (Additional file 2: S1(b)).

The different treatment strategies had different overall risks of bias, according to the standard made by Mutz et al. [45]. Among the tDCS monotherapy trials, two had low overall risk of bias and two had unclear overall risk of bias. For tDCS + medication trials, two had low and one had unclear overall risk of bias. Among the tDCS + psychotherapy trials, one had low, two had unclear, and one had high overall risk of bias. The only tDCS + ECT trial had a high overall risk of bias.

### Overall effects of tDCS treatments for depression

The results of the meta-analysis showed that the depression score of the active group was significantly lower compared to the sham group (*z* = 2.38, *p* = 0.017), the overall effect size of combing all therapies was small (Hedges’ *g* = − 0.442, 95% CI − 0.805 to − 0.079), while heterogeneity was moderate (*I*^2^ = 67.7%, *Q* = 37.18, *p* < 0.001). The overall dropout rates of the active and sham groups were 7.17% (18/251) and 11.8% (24/204), respectively. The difference in dropout rate between the active and sham groups was not significant (*z* = 1.48, *p* = 0.140), with a positive (favouring active treatment) overall effect size OR = 0.58 (95% CI 0.281–1.196) and no evidence suggesting heterogeneity (*I*^2^ = 0.0%, *Q* = 8.08, *p* = 0.621).

In terms of secondary outcomes, the overall response rate in the active group (41.95%, 99/236) was higher than that in the sham group (29.73%, 50/185). The difference in the response rate between the two groups was marginally significant (*z* = 1.85, *p* = 0.065), with a positive (favouring active treatment) overall effect size OR = 1.525 (95% CI 0.975–2.385) and no evidence suggesting heterogeneity (*I*^2^ = 0.0%, *Q* = 10.84, *p* = 0.457). The remission rate in the active group (22.46%, 53/236) was also higher than that in the sham group (16.22%, 30/185). The difference in the remission rate between the two groups was also non-significant (*z* = 0.72, *p* = 0.470), with a positive overall effect size OR = 1.265 (95% CI 0.669–2.395) and low heterogeneity (*I*^2^ = 15.2%, *Q* = 11.79, *p* = 0.299).

Furthermore, all four outcomes were gradually stable with a gradually narrow confidence interval over time, according to the results of cumulative meta-analysis (Additional file 3: Figure S2).

### Efficacy of different treatment strategies for depression

#### Severity

**Fig. 2:**
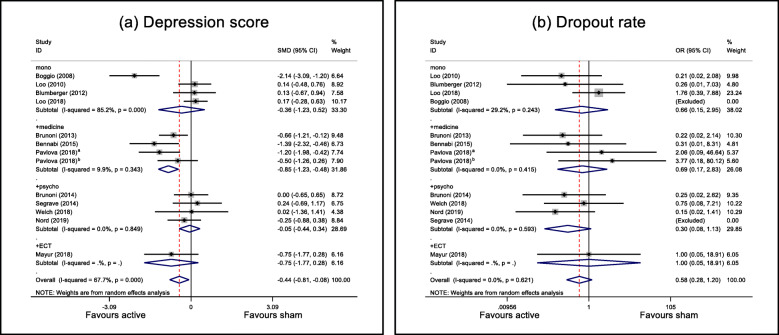
Forest plots of primary outcomes: **a** depression score, **b** dropout rate. mono = tDCS alone; +medicine = tDCS + medication; + psycho = tDCS + psychotherapy; +ECT = tDCS + ECT. SMD, standardised mean difference; OR, odds ratio; CI, confidence interval. ^a^Stimulation duration, 30 min. ^b^Stimulation duration, 20 min. “Excluded” in brackets means the events in both active and sham groups are zero, and thus, the study was excluded from further analysis

Severity was assessed by the depression score. We calculated the effect sizes of depression scores for the four different treatment strategies (i.e. tDCS alone, tDCS + medication, tDCS + psychotherapy and tDCS + ECT). Subgroup analysis (Fig. 2a) revealed that the superiority of active treatment was only present in the tDCS + medication therapy group (*g* = − 0.855, 95% CI − 1.234 to − 0.475, *z* = 4.42, *p* < 0.001), but not in the tDCS alone (*g* = − 0.358, 95% CI − 1.232 to 0.515, *z* = 0.80, *p* = 0.421), tDCS + psychotherapy (*g* = − 0.053, 95% CI − 0.443 to 0.337, *z* = 0.27, *p* = 0.790), or tDCS + ECT (*g* = − 0.746, 95% CI − 1.77 to 0.279, *z* = 1.43, *p* = 0.154) groups. High heterogeneity was only observed in the tDCS alone group (*I*^2^ = 85.2%, *Q* = 20.26, *p* < 0.001), but tDCS + medication (*I*^2^ = 9.9%, *Q* = 3.33, *p* = 0.343) and tDCS + psychotherapy (*I*^2^ = 0.0%, *Q* = 0.8, *p* = 0.849) therapies had low heterogeneities.

#### Acceptability

Acceptability was evaluated using the dropout rate. Subgroup analysis indicated that the dropout rates were similar in both groups for all treatment strategies: tDCS alone, OR = 0.656, 95% CI 0.164–2.949, *z* = 0.55, *p* = 0.583; tDCS + medication, OR = 0.688, 95% CI 0.167–2.835, *z* = 0.52, *p* = 0.604; tDCS + psychotherapy therapy, OR = 0.302, 95% CI 0.080–1.134, *z* = 1.77, *p* = 0.076; tDCS + ECT therapy, OR = 1.000, 95% CI 0.053–18.915, *z* = 0.00, *p* = 1.000. Heterogeneities of the tDCS alone, tDCS + medication, and tDCS + psychotherapy groups were all low and non-significant, with *I*^2^ = 29.2%, 0.0%, and 0.0%, respectively (Fig. 2b).

#### Response rate

**Fig. 3:**
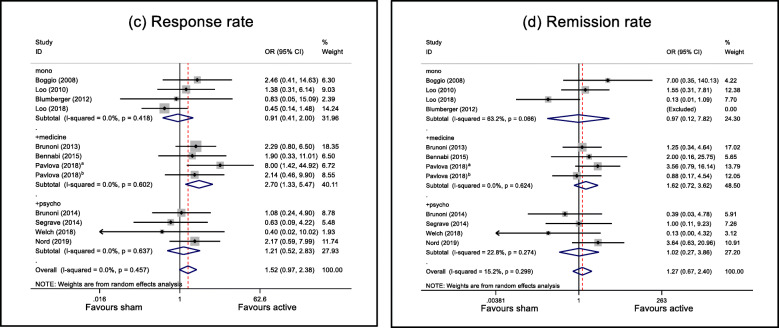
Forest plots of secondary outcomes: **a** response rate, **b** remission rate. mono = tDCS alone; +medicine = tDCS + medication; +psycho = tDCS + psychotherapy; +ECT = tDCS + ECT. SMD, standardised mean difference; OR, odds ratio; CI, confidence interval. ^a^Stimulation duration is 30 minutes. ^b^Stimulation duration is 20 min. “Excluded” in brackets means the events in both active and sham groups are zero, and thus, the study was excluded from further analysis

Subgroup analysis illustrated that the response rate of the active group was higher than that of the sham group only in the tDCS + medication therapy (OR = 2.700, 95% CI 1.332–5.471, *z* = 2.76, *p* = 0.006). The response rates were similar in active and sham groups for the other treatment strategies: tDCS alone, OR = 0.908 (95% CI 0.411–2.003, *z* = 0.24, *p* = 0.81) and tDCS + psychotherapy therapy (OR = 1.125, 95% CI 0.521–2.832, *z* = 0.45, *p* = 0.652). As shown in Fig. 3a, low and non-significant heterogeneities were observed not only overall, but also for tDCS alone (*I*^2^ = 0.0%, *Q* = 2.84, *p* = 0.418), tDCS + medication (*I*^2^ = 0.0%, *Q* = 1.86, *p* = 0.602) and tDCS + psychotherapy (*I*^2^ = 0.0%, *Q* = 1.7, *p* = 0.637).

#### Remission rate

Subgroup analysis showed that the remission rates were similar in active and sham groups for all treatment strategies: tDCS alone, OR = 0.968 (95% CI 0.120–7.817, *z* = 0.03, *p* = 0.976), tDCS + medication, OR = 1.620 (95% CI 0.724–3.625, *z* = 1.17, *p* = 0.241), and tDCS + psychotherapy, OR = 1.018, 95% CI 0.268–3.860, *z* = 0.03, *p* = 0.979). Low and non-significant heterogeneities were observed in the tDCS + medication (*I*^2^ = 0.0%, *Q* = 1.76, *p* = 0.624) and tDCS +psychotherapy (*I*^2^ = 22.8%, *Q* = 3.88, *p* = 0.274), but not in the tDCS alone (*I*^2^ = 63.2%, *Q* = 5.44, *p* = 0.066) (Fig. 3b).

### Influence of clinical characteristics and montage parameters

Meta-regression analyses provided no evidence of association between the depression score or dropout rate and clinical characteristics (i.e. publication year, baseline score, treatment strategy), demographics (i.e. sample size, age, and female proportion), and montage parameters (i.e. electrode size, current intensity, stimulation duration, number of sessions, and total charge). The details are shown in Additional file 4: Table S1. However, several variables have shown trends of association with response and remission rates (Table 3). A stepwise regression model was conducted between response rate and multiple variables, including tDCS alone, tDCS + medication, current intensity, number of sessions, and total charge. One significant model was found (*F*_(1, 10)_ = 6.69, *p* = 0.027, Adj R-squared = 0.34) with a significant contribution from only one variable: current intensity (*B* = − 1.90, 95% CI − 3.54 to − 0.26, *p* = 0.027). The model for the remission rate, consisting of number of sessions and total charge that were the significant factors found in univariate meta-regression models, showed no statistical significance.

**Table 3:** Univariable meta-regression

	Response rate			Remission rate		
	Coef (*B*)	95% CI	*p*	Coef (*B*)	95% CI	*p*
*Clinical characteristics*						
Publication year	− 0.12	− 0.18 to 0.16	0.873	− 0.05	− 0.29 to 0.18	0.629
Baseline score ^a^	− 0.06	− 0.18 to 0.05	0.232	− 0.07	− 0.20 to 0.7	0.279
Treatment strategy						
Monotherapy	− 1.09	− 2.31 to 0.13	**0.075**	− 0.58	− 2.66 to 1.49	0.536
tDCS +medication	0.95	− 0.08 to 1.99	**0.068**	0.49	− 1.04 to 2.01	0.489
tDCS +psychotherapy	− 0.799	− 2.07 to 0.47	0.189	− 0.44	− 2.43 to 1.55	0.624
*Demographics*						
Sample size	− 0.01	− 0.04 to 0.01	0.304	− 0.02	− 0.06 to 0.02	0.258
Age	− 0.04	− 0.12 to 0.05	0.359	− 0.05	− 0.17 to 0.08	0.422
Female rate	2.69	− 0.82 to 6.19	0.119	1.99	− 3.18 to 7.16	0.407
*Montage parameters*						
Size of electrode	− 0.06	− 0.14 to 0.02	0.114	− 0.01	− 0.11 to 0.10	0.912
Current intensity	− 0.73	− 1.48 to 0.02	**0.057**	− 0.74	− 1.68 to 0.21	0.111
Stimulation duration	− 0.19	− 0.14 to 0.1	0.723	− 0.07	− 0.23 to 0.09	0.335
Number of sessions	− 0.11	− 0.24 to 0.02	**0.090**	− 0.21	− 0.42 to 0.01	**0.060**
Total charge ^b^	− 0.03	− 0.07 to 0.00	**0.080**	− 0.06	− 0.12 to − 0.00	**0.040**

^a^Baseline score was calculated by the weighted arithmetic mean of depression scores of active and sham groups

^b^Total charge = (current intensity × stimulation duration × number of sessions)/size of electrode; boldface means *q* < 0.1

### Sensitivity analysis

The influence analysis with random models showed the distribution of the combined effect size after excluding each corresponding study. The combined effect size after excluding any study was similar to the overall effect size (Additional file 5: Figure S3). All confidence intervals were located around the overall 95% CI (− 0.74 to − 0.00) for the depression score, the overall 95% CI (0.28 to 1.20) for dropout rate, the overall 95% CI (0.97 to 2.38) for the response rate, and the overall 95% CI (0.67 to 2.40) for the remission rate. The detailed values of the estimated effect sizes and CIs for each study are shown in Additional file 5: Table S2.

Meta-analysis excluding trials at high risk of bias showed that the active group remained superior to the sham group in terms of overall depression score and response rate when considering only 10 RCTs without a high risk of bias, and the active group’s overall dropout and remission rates remained no superior to them in the sham group. The significance of three treatment strategies (i.e. tDCS alone, tDCS + medication, and tDCS + psychotherapy) also did not change after excluding high-risk trials for depression score, response, and remission rates. The only change was the dropout rate of tDCS + psychotherapy which changed into significant (*p* = 0.046) from original marginally significant (*p* = 0.076). Additional file 6: Figure S4 shows the forest plots. In summary, the results derived from the included trials were robust.

### Publication bias

The funnel plots of the depression score did not suggest small study effects (Additional file 6: Figure S5), which would have implied that smaller studies sometimes show different, often larger, treatment effects than large studies. Egger’s test of depression scores was not significant (*p* = 0.155). Specifically, further analysis confirmed that Egger’s tests for tDCS alone, tDCS + medication, and tDCS + psychotherapy treatments were all not significant. tDCS + ECT therapy was not analysed because this group included only one trial. There was no overall publication bias for dropout rate and response rate, and none of the three treatment strategies were biased (Additional file 6: Figure S5). However, the tDCS + psychotherapy group made a significant contribution to this bias (*p* = 0.037), although the remission rate manifested a non-significant bias (Additional file 6: Table S3).

## Discussion

In this study, we examined the effects of tDCS, alone and in combination with other therapies (i.e. medication, psychotherapy and ECT), on severity, acceptability, treatment response, and remission for major depressive disorder. We found that only tDCS + medication resulted in a significantly lower depression score and greater response rate than the sham condition. Potential influential factors on the four outcome measures were also investigated. Regression models showed that current intensity was the only factor that was significantly correlated with response rate. The sensitivity and publication bias analyses demonstrated that the results of the included 12 RCTs were robust and unbiased.

### Treatment with tDCS generally achieves weak effectiveness

According to the results of 12 randomised sham-controlled studies, the tDCS active groups showed a significant smaller depression score, similar dropout rate, and had marginally significantly higher response rate than the sham group at the immediate post-treatment endpoints, but the remission rates were not significantly different. Previous systematic reviews also showed consistent findings of significantly decreased depression scores and non-significant dropout rates, while findings for other outcomes were mixed. Some reviews reported non-significant results for response and remission [15, 26, 27], while others have shown significant results [28, 45, 53]. Although there were no salient differences found in this study, the active group presented better performance with fewer dropout events and higher response and remission events. Notably, the results of the meta-analysis suggested that tDCS had a limited curative effect, with high heterogeneity in treating adults with major depressive disorder. The weak effectiveness might be related to the limited number of sessions, since most studies so far only examined effects of 10–20 sessions, which is now considered insufficient to show antidepressant effects of tDCS [29].

### tDCS + medication shows superior therapeutic efficacy

Subgroup analysis showed that tDCS + medication has a significantly lower depression score and a higher response rate, with low heterogeneity for all four outcomes. This treatment had a large effect size (*g* = − 0.855) for depression score and response rate was 2.7 times greater than in the sham group. This finding is not only encouraging for clinical practice, as patients are thus not required to withdraw antidepressant use during tDCS treatment, but also plausible, as several other neuromodulation therapies require simultaneous medication intake, such as deep brain stimulation for Parkinson’s disease. This result is consistent with the previous predictive models of antidepressant response to active tDCS, suggesting that antidepressant medication is a stable variable that can identify patients more likely to show better tDCS antidepressant response [54]. It has been indicated in a few reviews [15, 26, 27] that combined tDCS and medication could result in a negative efficacy or non-superiority to sham for treating MDD. Meanwhile, many studies [31, 51, 55] and reviews [28, 33, 53] have shown a particular increase in antidepressant efficacy when tDCS co-initiated with or added to SSRIs. Similar to these studies, the medications prescribed in three of the tDCS + medication trials in this meta-analysis were two types of selective serotonin reuptake inhibitors (SSRIs), i.e. sertraline hydrochloride and escitalopram. To investigate the combining effects of tDCS and different medications to both unipolar and bipolar depression, our further analysis (Additional file 7: Table S4 & S4) showed that tDCS + SSRIs only [54] has an optimal effect compared against tDCS + mixed medications [14, 56–58]. The therapy of tDCS + SSRIs only showed consistently positive effects, significantly bigger improvement, and a higher response rate with a significant trend (Additional file 7: Figure. S6). Besides, studies only recruited patients with unipolar depression gained a significantly larger improvement than studies that included both uni- and bi- polar. Such comparison analysis provided further evidence for the findings of this meta-analysis that the combination of tDCS and SSRIs only provides effective treatment for unipolar depression (Additional file 7: S5).

The mechanisms underlying the effects of the tDCS and SSRIs combination therapy for depression could be due to bottom-up and top-down regulation of activation of brain circuits and/or reinforcement of serotonergic neurotransmission. Possibly, the additional benefits of the tDCS + SSRIs were driven by both bottom-up (SSRIs acts primarily on the downregulation of limbic subcortical hyperactivity) and top-down (tDCS acts primarily on frontal cortical activation) regulations, and in combination, these distinct functions jointly result in a faster and greater response [31]. Another possibility is that serotonergic reinforcement may enhance facilitatory aftereffects and thereby increase the efficacy of tDCS [59]. The study of Nitsche et al. [59] showed that serotonin’s impact on neuroplasticity might act by modulating the effects of citalopram (one of SSRIs) on plasticity induced by tDCS in humans and found that citalopram enhanced and prolonged the facilitation induced by anodal tDCS.

However, the effects of tDCS in combination with other medicines should be examined with caution. A mixed experimental outcome showed that tDCS treatment outcome varies depending on the type of medicine [60]. The combination of tDCS and benzodiazepines, mood stabilisers (e.g. carbamazepine), antipsychotics, or other medications (e.g. L-dopa, rivastigmine, dextromethorphan, and flunarizine) could decrease the anodal tDCS effects [60, 61] over both local and remote areas [62].

Other strategies did not show superior efficacies. tDCS + psychotherapy shown no obvious change in severity, response rate, and remission rate after treatment. This result is consistent with earlier systematic reviews that either stated that insufficient evidence to support this combination therapy [33] or found that adjunctive cognitive-control training negatively affected the treatment effect of tDCS [15]. The depression score and response rate of the only trial with tDCS + ECT were not salient. Surprisingly, in contrast with the previous review [27], tDCS mono had no significant effect on any of the four outcomes in this study, which might be due to its high heterogeneities in depression score and remission rate.

### Treatment strategy is a source of heterogeneity

Significant heterogeneity existed in the primary outcome “depression score”. Subgroup analyses revealed that treatment strategy is a crucial source of heterogeneity which is reflected in very varying heterogeneities in different strategies. Regression models indicated that most clinical characteristics and montage parameters did not contribute to the depression score, dropout rate, or remission rate. The only factor with an influence on outcomes was the current intensity which significantly contributed to the increased response rate. There was no significant correlation between dosage (total charge) and therapeutic effect, according to the results of stepwise regression models. In contrast, a significant positive association was found between accumulated dosage and therapeutic effect by Brunoni et al. [28]. This discrepancy may be due to the use of different definitions of dosage. The definition of Brunoni et al. was the mean dose per square centimetre, but we chose the accumulated total charge as the dosage (see the “6” section).

### Quality and robustness

The included studies are robust. The influence analyses indicated that the results of four outcomes (i.e. depression score, dropout rate, response rate, and remission rate) were robust, because there was no notable change in the combined effect size after excluding each study individually. Moreover, the results of the two excluded high-risk trials did not change the significance of the results of the 12 included RCTs.

Likewise, there was no evidence of overall publication bias for the four outcomes, and no publication bias for two treatment strategies tDCS alone and tDCS + medication, except for remission rate of tDCS + psychotherapy.

The quality of included studies is inconclusive. The risk of bias assessment revealed that 16.7%, 41.7%, and 41.7% of the trials were assessed as having high, unclear, and low risk of bias, respectively. The sources of unclear risk of bias involved random sequence generation, allocation concealment, blinding of participants and personnel, and outcome assessment. A relevant review [63] found significantly lower effect sizes in studies with unclear randomization methods than in studies with appropriate methods. In this study, two treatment strategies, tDCS alone and tDCS + medication, had lower risk of bias than the other two strategies, which suggested that the quality of studies reporting on tDCS alone and tDCS + medication was better.

## Limitations

Several limitations of our systematic review should be mentioned. First, the number of trials is small for each combination therapy and limits the generalization of our conclusions. Second, half of the included studies exhibited an unclear risk of bias, and the overall risk was deemed high in two trials (16.7%). Nevertheless, sensitivity analysis results showed that the original findings were unaltered after the exclusion of the two trials that had a high risk of bias. Third, treatment-resistant depression which could potentially influence treatment outcomes was not investigated, because the data in some trials were inaccessible. Treatment-resistant depression could be important, because some studies found that tDCS only affects non-treatment-resistant depression [20, 52]. Fourth,  high heterogeneity was found in the included studies. However, subgroup analyses indicated that treatment strategy was the main factor associated with the observed heterogeneity, and the heterogeneity was low within each treatment strategy group. Moreover, this study followed strict inclusion criteria to ensure that the findings would be more conclusive. Fifth, strict inclusion and exclusion criteria could increase homogeneity among studies but would also decrease the number of trials. In this study, one important RCT [64] and two arms in the study of Brunoni et al. [31] were excluded due to included patients with bipolar disorder and added with placebo medication, respectively, which decreased the number of monotherapies and might be further led to the negative result of monotherapy on MDD. Therefore, the trade-off between criteria and trials’ number should be very careful in future studies. Sixth is the categorization issue. The different definitions of treatment strategy might lead to a different conclusion from previous studies. The combination therapy was defined as “add-on therapy” (e.g. [26]), “mixed therapy” (e.g. [20, 27]), and “augmentation therapy” (e.g. [20, 26, 27]) in previous meta-analysis articles. However, these definitions permit to merge of different therapies like medication and psychotherapy into a whole that resulted in a mixed and unexplainable result. It is hard to draw generalizable conclusions. Therefore, we decided to categorize treatment strategies according to the therapy each study utilized. However, our categorization also caused problems and need to be optimized. For tDCS + medication therapy, we only included the studies that stated all their participants took unified medicine, and excluded varying prescriptions, adding placebo and non-unipolar populations. Therefore, Loo et al. [12, 32] and Blumberger et al. [52] were categorized into monotherapy due to complex situations of medication, such as a part of participants were medication-free. Additionally, merging cognitive control training and cognitive behaviour therapy into tDCS + psychotherapy might not be reasonable, although the heterogeneity seems low. These approaches probably caused the contradictory result from prior meta-analyses (e.g. [26]) on this topic.

## Conclusions

In conclusion, this systematic review and meta-analysis provides promising evidence that the combination of tDCS and antidepressant medication could achieve significant clinical improvement for adults with unipolar depression. Our results indicated that the combination of tDCS and SSRIs might be most effective for decreasing depression scores and increasing response rates, with low heterogeneities. This finding is encouraging for clinical practice, as patients are thus not required to withdraw antidepressant use during tDCS treatment. It would be important and valuable to further confirm the effects of a combination of tDCS and SSRIs with meticulously designed, large-scale multi-centre RCT studies. While this study investigated the post-acute effects, future research should also investigate the long-term effects.

## Supplementary Information

**Additional file 1: S1.** The list of excluded studies by full-text assessment and the reasons. **S2.** The list of included studies.

**Additional file 2: S3.** Supporting statements. **Figure S1.** Risk of bias.

**Additional file 3: Figure S2.** Cumulative meta-analysis.

**Additional file 4: Table S1.** Univariable meta-regression for score and acceptability.

**Additional file 5: Figure S3.** Influence analyses with random models (a) depression score by Hedges; (b) acceptability by OR; (c) response by OR; (d) remission by OR. **Table S2.** Results of influence analyses with random models (a) primary outcomes; (b) secondary outcomes. **Figure S4.** Meta-analysis (excluded high risk trials).

**Additional file 6: Figure S5.** Funnel plots by treatment strategy (a) depression score; (b) acceptability; (c) response rate; (d) remission rate. **Table S3.** Egger’s test for each outcome.

**Additional file 7: Table S4.** study characteristics for each trial with tDCS + medication therapy. **S4.** The list of included studies: tDCS + medication therapy. **S5.** Further investigation of tDCS + medication therapy. **Figure S6.** The influence of therapies of tDCS plus SSRIs only or SSRIs with other medications on unipolar depression or bipolar disorder.

## Data Availability

The data used and/or analysed during the current study are available in the manuscript and Additional files, except for the values of outcome measures that could be available only from the corresponding author on reasonable request.

## Abbreviations

CI: Confidence interval
dlPFC: Dorsal lateral prefrontal cortex
ECT: Electroconvulsive therapy
HDRS: Hamilton Depression Rating Scale
MADRS: Montgomery–Åsberg Depression Rating Scale
MDD: Major depressive disorder
OR: Odds ratio
RCT: Randomized controlled trial
SSRI: Selective serotonin reuptake inhibitor
tDCS: Transcranial direct current stimulation
