# Supplementary material for: Is transcranial direct current stimulation, alone or in combination with antidepressant medications or psychotherapies, effective in treating major depressive disorder? A systematic review and meta-analysis
Source: BMC Med. 2021 Dec 17;19:319. doi: 10.1186/s12916-021-02181-4 (PMC8680114; doi:10.1186/s12916-021-02181-4)
Supplement: Supplementary file 1 — Additional file 1: S1. The list of excluded studies by full-text assessment and the reasons. S2. The list of included studies. [file 12916_2021_2181_MOESM1_ESM.docx]

# Included and excluded publications

## S1. The list of excluded studies by full-text assessment and the reasons

| **Publication** | **Exclusion**  **Reason** |
| --- | --- |
| 1. Al-Kaysi, A. M., Al-Ani, A., Loo, C. K., Breakspear, M., & Boonstra, T. W. (2016). Predicting brain stimulation treatment outcomes of depressed patients through the classification of EEG oscillations. Conf Proc IEEE Eng Med Biol Soc, 2016, 5266-5269. doi:10.1109/embc.2016.7591915 | ➀ |
| 1. Al-Kaysi, A. M., Al-Ani, A., Loo, C. K., Powell, T. Y., Martin, D. M., Breakspear, M., & Boonstra, T. W. (2017). Predicting tDCS treatment outcomes of patients with major depressive disorder using automated EEG classification. J Affect Disord, 208, 597-603. doi:10.1016/j.jad.2016.10.021 | ➀ |
| 1. Alonzo, A., Chan, G., Martin, D., Mitchell, P. B., & Loo, C. (2013). Transcranial direct current stimulation (tDCS) for depression: analysis of response using a three-factor structure of the Montgomery-Asberg depression rating scale. J Affect Disord, 150(1), 91-95. doi:10.1016/j.jad.2013.02.027 | ➄ |
| 1. Alonzo, A., Aaronson, S., Bikson, M., Husain, M., Lisanby, S., Martin, D., . . . Loo, C. (2016). Study design and methodology for a multicentre, randomised controlled trial of transcranial direct current stimulation as a treatment for unipolar and bipolar depression. Contemp Clin Trials, 51, 65-71. doi:10.1016/j.cct.2016.10.002 | ➀ |
| 1. Alonzo, A., Fong, J., Ball, N., Martin, D., Chand, N., & Loo, C. (2019). Pilot trial of home-administered transcranial direct current stimulation for the treatment of depression. *Journal of Affective Disorders*, *252*, 475–483. | ➁ |
| 1. Aparicio, L. V. M., Rosa, V., Razza, L. M., Sampaio-Junior, B., Borrione, L., Valiengo, L., . . . Brunoni, A. R. (2019). Transcranial direct current stimulation (tDCS) for preventing major depressive disorder relapse: Results of a 6-month follow-up. Depression and Anxiety, 36(3), 262-268. doi:http://dx.doi.org/10.1002/da.22878 | ➁ |
| 1. Bai, S., Dokos, S., Ho, K. A., & Loo, C. (2014). A computational modelling study of transcranial direct current stimulation montages used in depression. Neuroimage, 87, 332-344. https://doi.org/10.1016/j.neuroimage.2013.11.015 | ➀ |
| 1. Bajbouj, M., Aust, S., Spies, J., Herrera-Melendez, A. L., Mayer, S. V., Peters, M., . . . Padberg, F. (2018). PsychotherapyPlus: augmentation of cognitive behavioral therapy (CBT) with prefrontal transcranial direct current stimulation (tDCS) in major depressive disorder-study design and methodology of a multicenter double-blind randomized placebo-controlled trial. Eur Arch Psychiatry Clin Neurosci, 268(8), 797-808. doi:10.1007/s00406-017-0859-x | ➀ |
| 1. Bares M, Brunovsky M, Stopkova P, Hejzlar M, Novak T. Transcranial Direct-Current Stimulation (tDCS) Versus Venlafaxine ER in The Treatment of Depression: A Randomized, Double-Blind, Single-Center Study with Open-Label, Follow-Up. Neuropsychiatr Dis Treat. 2019; 15:3003-3014. https://doi.org/10.2147/NDT.S226577 | ⑧ |
| 1. Bautovich, A., Loo, C., Katz, I., Martin, D., & Harvey, S. (2016). Transcranial Direct Current Stimulation as a Treatment for Depression in the Hemodialysis Setting. Psychosomatics, 57(3), 305-309. doi:10.1016/j.psym.2015.11.006 | ➁ |
| 1. Boggio, P. S., Bermpohl, F., Vergara, A. O., Muniz, A. L., Nahas, F. H., Leme, P. B., . . . Fregni, F. (2007). Go-no-go task performance improvement after anodal transcranial DC stimulation of the left dorsolateral prefrontal cortex in major depression. J Affect Disord, 101(1-3), 91-98. doi:10.1016/j.jad.2006.10.026 | ➅➆ |
| 1. Brennan, S., McLoughlin, D. M., O'Connell, R., Bogue, J., O'Connor, S., McHugh, C., & Glennon, M. (2017). Anodal transcranial direct current stimulation of the left dorsolateral prefrontal cortex enhances emotion recognition in depressed patients and controls. Journal of Clinical and Experimental Neuropsychology, 39(4), 384-395. https://doi.org/310.1080/13803395.13802016.11230595. | ➆ |
| 1. Brunoni, A. R., Ferrucci, R., Bortolomasi, M., Vergari, M., Tadini, L., Boggio, P. S., . . . Priori, A. (2011). Transcranial direct current stimulation (tDCS) in unipolar vs. bipolar depressive disorder. Progress in Neuro-Psychopharmacology and Biological Psychiatry, 35(1), 96-101. http://www.sciencedirect.com/science/article/pii/S0278584610003611. | ➁➃ |
| 1. Brunoni, Andre R., R. Ferrucci, M., Bortolomasi, E. Scelzo, Boggio, P. S., Fregni, F., . . . A. Priori (2013). Interactions between transcranial direct current stimulation (tDCS) and pharmacological interventions in the Major Depressive Episode: Findings from a naturalistic study. European Psychiatry, 28(6), 356-361. | ➂➃➄ |
| 1. Brunoni, A. R., Junior, R. F., Kemp, A. H., Lotufo, P. A., Bensenor, I. M., & Fregni, F. (2014). Differential improvement in depressive symptoms for tDCS alone and combined with pharmacotherapy: an exploratory analysis from the Sertraline vs. Electrical Current Therapy for Treating Depression Clinical Study. Int J Neuropsychopharmacol, 17(1), 53-61. doi:10.1017/s1461145713001065 | ➄ |
| 1. Brunoni, A. R., Schestatsky, P., Lotufo, P. A., Bensenor, I. M., & Fregni, F. (2014). Comparison of blinding effectiveness between sham tDCS and placebo sertraline in a 6-week major depression randomized clinical trial. Clin Neurophysiol, 125(2), 298-305. doi:10.1016/j.clinph.2013.07.020 | ➄ |
| 1. Brunoni, A. R., Machado-Vieira, R., Sampaio-Junior, B., Vieira, E. L., Valiengo, L., Bensenor, I. M., . . . Teixeira, A. L. (2015). Plasma levels of soluble TNF receptors 1 and 2 after tDCS and sertraline treatment in major depression: Results from the SELECT-TDCS trial. J Affect Disord, 185, 209-213. doi:10.1016/j.jad.2015.07.006 | ➀ |
| 1. Brunoni, A. R., Machado-Vieira, R., Zarate, C. A., Jr., Vieira, E. L., Valiengo, L., Bensenor, I. M., . . . Teixeira, A. L. (2015). Assessment of non-BDNF neurotrophins and GDNF levels after depression treatment with sertraline and transcranial direct current stimulation in a factorial, randomized, sham-controlled trial (SELECT-TDCS): an exploratory analysis. Prog Neuropsychopharmacol Biol Psychiatry, 56, 91-96. doi:10.1016/j.pnpbp.2014.08.009 | ➀ |
| 1. Brunoni, A. R., Sampaio-Junior, B., Moffa, A. H., Borrione, L., Nogueira, B. S., Aparicio, L. V., . . . Bensenor, I. M. (2015). The Escitalopram versus Electric Current Therapy for Treating Depression Clinical Study (ELECT-TDCS): rationale and study design of a non-inferiority, triple-arm, placebo-controlled clinical trial. Sao Paulo Med J, 133(3), 252-263. doi:10.1590/1516-3180.2014.00351712 | ➀ |
| 1. Brunoni, André Russowsky, Tortella, G., Benseñor, I. M., Lotufo, P. A., Carvalho, A. F., & Fregni, F. (2016). Cognitive effects of transcranial direct current stimulation in depression: Results from the SELECT-TDCS trial and insights for further clinical trials. *Journal of Affective Disorders*, *202*, 46–52. | ➄ |
| 1. Brunoni, Andre R., Moffa, A. H., Sampaio-Junior, B., Borrione, L., Moreno, M. L., Fernandes, R. A., Veronezi, B. P., Nogueira, B. S., Aparicio, L. V. M., Razza, L. B., Chamorro, R., Tort, L. C., Fraguas, R., Lotufo, P. A., Gattaz, W. F., Fregni, F., & Benseñor, I. M. (2017). *Trial of Electrical Direct-Current Therapy versus Escitalopram for Depression*. The New England Journal of Medicine. 376: 2523-2533. https://doi.org/10.1056/NEJMoa1612999 | ⑧ |
| 1. Brunoni, A. R., Padberg, F., Vieira, E. L. M., Teixeira, A. L., Carvalho, A. F., Lotufo, P. A., . . . Bensenor, I. M. (2018). Plasma biomarkers in a placebo-controlled trial comparing tDCS and escitalopram efficacy in major depression. Prog Neuropsychopharmacol Biol Psychiatry, 86, 211-217. doi:10.1016/j.pnpbp.2018.06.003 | ➀ |
| 1. Bulubas, L., Padberg, F., Bueno, P. V., Duran, F., Busatto, G., Amaro, E., . . . Brunoni, A. R. (2019). Antidepressant effects of tDCS are associated with prefrontal gray matter volumes at baseline: Evidence from the ELECT-TDCS trial. Brain Stimulation. doi:http://dx.doi.org/10.1016/j.brs.2019.05.006 | ➀ |
| 1. Chan, H. N., Alonzo, A., Martin, D. M., Mitchell, P. B., Sachdev, P., & Loo, C. K. (2013). Augmenting transcranial direct current stimulation with (D)-cycloserine for depression: a pilot study. J ect, 29(3), 196-200. doi:10.1097/YCT.0b013e3182801b09 | ➁➂➃ |
| 1. Chrysikou, E. G., Wing, E. K., & van Dam, W. O. (2019). Transcranial Direct Current Stimulation Over Prefrontal Cortex in Depression Modulates Cortical Excitability in Emotion Regulation Regions as Measured by Concurrent Functional Magnetic Resonance Imaging: An Exploratory Study. Biological Psychiatry: Cognitive Neuroscience and Neuroimaging. doi:10.1016/j.bpsc.2019.12.004 | ➅ |
| 1. Csifcsák, G., Boayue, N. M., Puonti, O., Thielscher, A., & Mittner, M. (2018). Effects of transcranial direct current stimulation for treating depression: A modeling study. Journal of Affective Disorders, 234, 164-173. http://www.sciencedirect.com/science/article/pii/S0165032717324746. | ➀ |
| 1. Dastjerdi, G., Mirhoseini, H., & Mohammadi, E. (2015). Investigating the synergistic effects of transcranial direct current stimulation and cranial electrical stimulation in treatment of major depression in a double blinded controlled trial. Biomedical and Pharmacology Journal, 8(2), 1267-1274. doi:http://dx.doi.org/10.13005/bpj/885 | ➃ |
| 1. Dell’Osso, B., Zanoni, S., Ferrucci, R., Vergari, M., Castellano, F., D’Urso, N., . . . Altamura, A. C. (2012). Transcranial direct current stimulation for the outpatient treatment of poor-responder depressed patients. European Psychiatry, 27(7), 513-517. http://www.sciencedirect.com/science/article/pii/S0924933811000332. | ➁➂➃ |
| 1. Dell'Osso, B., Dobrea, C., Arici, C., Benatti, B., Ferrucci, R., Vergari, M., . . . Altamura, A. C. (2014). Augmentative transcranial direct current stimulation (tDCS) in poor responder depressed patients: a follow-up study. CNS Spectr, 19(4), 347-354. doi:10.1017/s1092852913000497 | ➁➂➃ |
| 1. Ferrucci, R., Bortolomasi, M., Vergari, M., Tadini, L., Salvoro, B., Giacopuzzi, M., . . . Priori, A. (2009). Transcranial direct current stimulation in severe, drug-resistant major depression. Journal of Affective Disorders, 118(1), 215-219. http://www.sciencedirect.com/science/article/pii/S0165032709000822. | ➃➂ |
| 1. Ferrucci R, Bortolomasi M, Brunoni AR, Vergares M, Tadini L, Giacopuzzi M, et al. (2009). Comparative benefits of transcranial direct current stimulation (tDCS) treatment in patients with mild/moderate vs. severe depression. Clin Neuropsychiatry. 6(6), 246–51. | ➁➃ |
| 1. Fregni, F., Boggio, P. S., Nitsche, M. A., Marcolin, M. A., Rigonatti, S. P., & Pascual‐Leone, A. (2006). Treatment of major depression with transcranial direct current stimulation. Bipolar Disorders, 8(2), 203-204. https://onlinelibrary.wiley.com/doi/abs/210.1111/j.1399-5618.2006.00291.x. | ➆ |
| 1. Fregni, F., Boggio, P. S., Nitsche, M. A., Rigonatti, S. P., & Pascual‐Leone, A. (2006). Cognitive effects of repeated sessions of transcranial direct current stimulation in patients with depression. Depression and Anxiety, 23(8), 482-484 %* ©2006 Wiley‐Liss, Inc. https://onlinelibrary.wiley.com/doi/abs/2010.1002/da.20201. | ➆ |
| 1. Khayyer, Z., Ngaosuvan, L., Sikstrom, S., & Ghaderi, A. H. (2018). Transcranial direct current stimulation based on quantitative electroencephalogram combining positive psychotherapy for major depression. J Integr Neurosci, 17(2), 89-96. doi:10.31083/jin-170045 | ➃➆ |
| 1. Li, M.-S., Du, X.-D., Chu, H.-C., Liao, Y.-Y., Pan, W., Li, Z., & Hung, G. C.-L. (2019). Delayed effect of bifrontal transcranial direct current stimulation in patients with treatment-resistant depression: a pilot study. BMC Psychiatry, 19(1). doi:10.1186/s12888-019-2119-2 | ➂➃ |
| 1. Loo, C. K., Alonzo, A., Martin, D., Mitchell, P. B., Galvez, V., & Sachdev, P. (2012). Transcranial direct current stimulation for depression: 3-week, randomised, sham-controlled trial. The British Journal of Psychiatry, 200(1), 52-59. | ➂ |
| 1. Martin, D. M., Alonzo, A., Mitchell, P. B., Sachdev, P., Gálvez, V., & Loo, C. K. (2011). Fronto-extracephalic transcranial direct current stimulation as a treatment for major depression: An open-label pilot study. Journal of Affective Disorders, 134(1), 459-463. http://www.sciencedirect.com/science/article/pii/S0165032711002643. | ➁➆ |
| 1. Martin, D. M., Alonzo, A., Ho, K.-A., Player, M., Mitchell, P. B., Sachdev, P., & Loo, C. K. (2013). Continuation transcranial direct current stimulation for the prevention of relapse in major depression. Journal of Affective Disorders, 144(3), 274-278. http://www.sciencedirect.com/science/article/pii/S016503271200691X. | ➁➃ |
| 1. Martin, D. M., Yeung, K., & Loo, C. K. (2016). Pre-treatment letter fluency performance predicts antidepressant response to transcranial direct current stimulation. J Affect Disord, 203, 130-135. doi:10.1016/j.jad.2016.05.072 | ➀➂ |
| 1. Martin, D. M., Teng, J. Z., Lo, T. Y., Alonzo, A., Goh, T., Iacoviello, B. M., . . . Loo, C. K. (2018). Clinical pilot study of transcranial direct current stimulation combined with Cognitive Emotional Training for medication resistant depression. Journal of Affective Disorders, 232, 89-95. http://www.sciencedirect.com/science/article/pii/S0165032717318657. | ➁ |
| 1. Martin, D. M., McClintock, S. M., Aaronson, S. T., Alonzo, A., Husain, M. M., Lisanby, S. H., . . . Loo, C. K. (2018). Pre-treatment attentional processing speed and antidepressant response to transcranial direct current stimulation: Results from an international randomized controlled trial. Brain Stimulation. 11(6), 1282-1290. https://doi.org/10.1016/j.brs.2018.08.011 | ➄ |
| 1. Martin, D. M., Moffa, A., Nikolin, S., Bennabi, D., Brunoni, A. R., Flannery, W., . . . Loo, C. K. (2018). Cognitive effects of transcranial direct current stimulation treatment in patients with major depressive disorder: An individual patient data meta-analysis of randomised, sham-controlled trials. Neurosci Biobehav Rev, 90, 137-145. doi:10.1016/j.neubiorev.2018.04.008 | ➀ |
| 1. McClintock, S. M., Martin, D. M., Lisanby, S. H., Alonzo, A., McDonald, W. M., Aaronson, S. T., … Loo, C. K. (2020). Neurocognitive effects of transcranial direct current stimulation (tDCS) in unipolar and bipolar depression: Findings from an international randomized controlled trial. Depression and Anxiety. doi:10.1002/da.22988 | ➄➆ |
| 1. Monnart A, Vanderhasselt M, Schroder E, Campanella S, Fontaine P and Kornreich C (2019). Treatment of Resistant Depression: A Pilot Study Assessing the Efficacy of A tDCS-Mindfulness Program Compared to A tDCS-Relaxation Program. Front. Psychiatry 10:730. doi:10.3389/fpsyt.2019.00730 | ➃ |
| 1. Moreno, M. L., Goerigk, S. A., Bertola, L., Suemoto, C. K., Razza, L. B., Moffa, A. H., … Brunoni, A. R. (2019). Cognitive changes after tDCS and escitalopram treatment in major depressive disorder: results from the placebo-controlled ELECT-TDCS trial. Journal of Affective Disorders. doi:10.1016/j.jad.2019.12.009 | ➄ |
| 1. Nikolin, S., Martin, D., Loo, C. K., Iacoviello, B. M., & Boonstra, T. W. (2019). Assessing neurophysiological changes associated with combined transcranial direct current stimulation and cognitive emotional training for treatment‐resistant depression. European Journal of Neuroscience. doi:10.1111/ejn.14656 | ➃ |
| 1. Palm, U., Schiller, C., Fintescu, Z., Obermeier, M., Keeser, D., Reisinger, E., Pogarell, O., Nitsche, M. A., Möller, H.-J., & Padberg, F. (2012). Transcranial direct current stimulation in treatment resistant depression: A randomized double-blind, placebo-controlled study. Brain Stimulation, 5(3), 242–251. https://doi.org/10.1016/j.brs.2011.08.005 | ➂ |
| 1. Palm, U., Goerigk, S., Kirsch, B., Bäumler, L., Sarubin, N., Hasan, A., Brunoni, AR., Padberg, F. (2019). Treatment of major depression with a two-step tDCS protocol add-on to SSRI: Results from a naturalistic study. Brain stimulation. 12(1), 195-197. doi: 10.1016/j.brs.2018.10.003 | ➃ |
| 1. Park, C. H., Chang, W. H., Park, J. Y., Shin, Y. I., Kim, S. T., & Kim, Y. H. (2013). Transcranial direct current stimulation increases resting state interhemispheric connectivity. Neuroscience Letters, 539, 7-10. doi:http://dx.doi.org/10.1016/j.neulet.2013.01.047 | ➀ |
| 1. Park, S., Choi, W.-J., Kim, S., Kim, B., Son, S. J., Roh, D., … Park, J. Y. (2020). Effects of transcranial direct current stimulation using miniaturized devices vs sertraline for depression in Korea: A 6 week, multicenter, randomized, double blind, active-controlled study. Journal of Psychiatric Research. doi:10.1016/j.jpsychires.2020.04.012 | ➃ |
| 1. Player, M. J., Taylor, J. L., Weickert, C. S., Alonzo, A., Sachdev, P. S., Martin, D., . . . Loo, C. K. (2014). Increase in PAS-induced neuroplasticity after a treatment course of transcranial direct current stimulation for depression. J Affect Disord, 167, 140-147. doi:10.1016/j.jad.2014.05.063 | ➅➆ |
| 1. Salehinejad, M. A., Ghanavai, E., Rostami, R., & Nejati, V. (2017). Cognitive control dysfunction in emotion dysregulation and psychopathology of major depression (MD): Evidence from transcranial brain stimulation of the dorsolateral prefrontal cortex (DLPFC). J Affect Disord, 210, 241-248. doi:10.1016/j.jad.2016.12.036 | ➆ |
| 1. Shahsavar, Y., Ghoshuni, M., & Talaei, A. (2018). Quantifying clinical improvements in patients with depression under the treatment of transcranial direct current stimulation using event related potentials. Australas Phys Eng Sci Med, 41(4), 973-983. doi:10.1007/s13246-018-0696-x | ➁➆ |
| 1. Valiengo, L., Bensenor, I. M., Goulart, A. C., de Oliveira, J. F., Zanao, T. A., Boggio, P. S., . . . Brunoni, A. R. (2013). The sertraline versus electrical current therapy for treating depression clinical study (select-TDCS): results of the crossover and follow-up phases. Depress Anxiety, 30(7), 646-653. doi:10.1002/da.22079 | ➁➄➆ |
| 1. Vanderhasselt, M.-A., De Raedt, R., Namur, V., Lotufo, P. A., Bensenor, I. M., Boggio, P. S., & Brunoni, A. R. (2015). Transcranial electric stimulation and neurocognitive training in clinically depressed patients: A pilot study of the effects on rumination. *Progress in Neuro-Psychopharmacology and Biological Psychiatry*, *57*, 93–99. | ➆ |
| 1. Vanderhasselt, M. A., De Raedt, R., Namur, V., Valiengo, L. C. L., Lotufo, P. A., Bensenor, I. M., . . . Brunoni, A. R. (2016). Emotional reactivity to valence-loaded stimuli are related to treatment response of neurocognitive therapy. J Affect Disord, 190, 443-449. doi:10.1016/j.jad.2015.10.022 | ➆ |
| 1. Wolkenstein, L., & Plewnia, C. (2013). Amelioration of Cognitive Control in Depression by Transcranial Direct Current Stimulation. Biological Psychiatry, 73(7), 646-651. http://www.sciencedirect.com/science/article/pii/S0006322312008980. | ➂➅ |
| 1. Wolkenstein, L., Zeiller, M., Kanske, P., & Plewnia, C. (2014). Induction of a depression-like negativity bias by cathodal transcranial direct current stimulation. Cortex, 59, 103-112. doi:10.1016/j.cortex.2014.07.011 | ➆ |
| 1. Zanao, T. A., Moffa, A. H., Shiozawa, P., Lotufo, P. A., Bensenor, I. M., & Brunoni, A. R. (2014). Impact of two or less missing treatment sessions on tDCS clinical efficacy: results from a factorial, randomized, controlled trial in major depression. Neuromodulation, 17(8), 737-742; discussion 742. doi:10.1111/ner.12167 | ➄ |
| 1. Zhou, Q., Yu, C., Yu, H., Zhang, Y., Liu, Z., Hu, Z., … Zhou, D. (2020). The Effects of Repeated Transcranial Direct Current Stimulation on Sleep Quality and Depression symptoms in Patients with Major Depression and Insomnia. Sleep Medicine. doi:10.1016/j.sleep.2020.02.003 | ➆ |

Note: ➀non-intervention study; ➁non-RCT study; ➂included bipolar depressive patients and cannot be separated from the sample; ➃lack of sham-control group; ➄ duplicative dataset from the same trial; ➅session<5; ➆lack of main outcomes; ⑧cannot exclude the placebo effect.

## S2. The list of included studies

1. Blumberger, D., Tran, L., Fitzgerald, P., Hoy, K. B., & Daskalakis, Z. J. (2012). A randomized double-blind sham-controlled study of transcranial direct current stimulation for treatment-resistant major depression. Frontiers in psychiatry, 3, 74.
2. Bennabi, D., Nicolier, M., Monnin, J., Tio, G., Pazart, L., Vandel, P., & Haffen, E. (2015). Pilot study of feasibility of the effect of treatment with tDCS in patients suffering from treatment-resistant depression treated with escitalopram. Clin Neurophysiol, 126(6), 1185-1189. doi:10.1016/j.clinph.2014.09.026
3. Boggio, P. S., Rigonatti, S. P., Ribeiro, R. B., Myczkowski, M. L., Nitsche, M. A., Pascual-Leone, A., & Fregni, F. (2008). A randomized, double-blind clinical trial on the efficacy of cortical direct current stimulation for the treatment of major depression. Int J Neuropsychopharmacol, 11(2), 249-254. doi:10.1017/s1461145707007833
4. Brunoni, A. R., Boggio, P. S., De Raedt, R., Bensenor, I. M., Lotufo, P. A., Namur, V., . . . Vanderhasselt, M. A. (2014). Cognitive control therapy and transcranial direct current stimulation for depression: a randomized, double-blinded, controlled trial. J Affect Disord, 162, 43-49. doi:10.1016/j.jad.2014.03.026
5. Brunoni, A. R., Valiengo, L., Baccaro, A., Zanao, T. A., de Oliveira, J. F., Goulart, A., . . . Fregni, F. (2013). The sertraline vs. electrical current therapy for treating depression clinical study: results from a factorial, randomized, controlled trial. JAMA Psychiatry, 70(4), 383-391. doi:10.1001/2013.jamapsychiatry.32
6. Loo, C. K., Husain, M. M., McDonald, W. M., Aaronson, S., O'Reardon, J. P., Alonzo, A., . . . Lisanby, S. H. (2018). International randomized-controlled trial of transcranial Direct Current Stimulation in depression. Brain Stimul, 11(1), 125-133. doi:10.1016/j.brs.2017.10.011
7. Loo, C. K., Sachdev, P., Martin, D., Pigot, M., Alonzo, A., Malhi, G. S., . . . Mitchell, P. (2010). A double-blind, sham-controlled trial of transcranial direct current stimulation for the treatment of depression. Int J Neuropsychopharmacol, 13(1), 61-69. doi:10.1017/s1461145709990411
8. Mayur, P., Howari, R., Byth, K., & Vannitamby, R. (2018). Concomitant Transcranial Direct Current Stimulation With Ultrabrief Electroconvulsive Therapy: A 2-Week Double-Blind Randomized Sham-Controlled Trial. J ect, 34(4), 291-295. doi:10.1097/yct.0000000000000479
9. Nord, C. L., Halahakoon, D. C., Limbachya, T., Charpentier, C., Lally, N., Walsh, V., . . . Roiser, J. P. (2019). Neural predictors of treatment response to brain stimulation and psychological therapy in depression: a double-blind randomized controlled trial. Neuropsychopharmacology, 44(9), 1613-1622. doi:http://dx.doi.org/10.1038/s41386-019-0401-0
10. Pavlova, E. L., Menshikova, A. A., Semenov, R. V., Bocharnikova, E. N., Gotovtseva, G. N., Druzhkova, T. A., . . . Guekht, A. B. (2018). Transcranial direct current stimulation of 20- and 30-minutes combined with sertraline for the treatment of depression. Prog Neuropsychopharmacol Biol Psychiatry, 82, 31-38. doi:10.1016/j.pnpbp.2017.12.004
11. Segrave, R. A., Arnold, S., Hoy, K., & Fitzgerald, P. B. (2014). Concurrent cognitive control training augments the antidepressant efficacy of tDCS: a pilot study. Brain Stimul, 7(2), 325-331. doi:10.1016/j.brs.2013.12.008
12. Welch, E. S., Weigand, A., Hooker, J. E., Philip, N. S., Tyrka, A. R., Press, D. Z., & Carpenter, L. L. (2018). Feasibility of Computerized Cognitive-Behavioral Therapy Combined with Bifrontal Transcranial Direct Current Stimulation for Treatment of Major Depression. Neuromodulation: Technology at the Neural Interface. International Neuromodulation Society. *0*(0). doi.org/10.1111/ner.12807.
