# Supplementary material for: Is transcranial direct current stimulation, alone or in combination with antidepressant medications or psychotherapies, effective in treating major depressive disorder? A systematic review and meta-analysis
Source: BMC Med. 2021 Dec 17;19:319. doi: 10.1186/s12916-021-02181-4 (PMC8680114; doi:10.1186/s12916-021-02181-4)

# Risk of bias

## S3. Supporting statements

|  | **Random sequence generation (selection bias)** | **Allocation concealment (selection bias)** | **Blinding of participants and personnel (performance bias)** | **Blinding of outcome assessment (detection bias)** | **Incomplete outcome data**  **(attrition bias)** | **Selective reporting (reporting bias)** |
| --- | --- | --- | --- | --- | --- | --- |
| Boggio, 2008 | “Randomization was performed using the order of entrance in the study and a previous randomization list generated by computer.” | no information. | “This device has a special feature that makes it particularly reliable for double blind trials.”  “We therefore incorporated a switch in the back of the tDCS device that could be activated by the researcher to interrupt the electrical current while maintaining the display ‘ON’ and displaying the parameters of stimulation throughout the procedure.” | “Rating was performed by a trained experienced psychologist (M.L.M.) blinded to the patients’ treatment group assignment.” | “There were no dropouts, and the few missing data (< 3% of the data) were considered at random.” | Results of all primary outcome measures were reported. |
| Loo, 2010 | “Subjects were stratified by age and gender and then randomly assigned to active or sham treatment groups.” | no information | “with raters and subjects blind to treatment group assignment.”  “After the ten sessions, the integrity of the blinding was assessed by asking subjects to guess whether they had been assigned to the active or sham treatment group.” “When asked to guess their treatment group at the end of the ten-session double blind phase, […]. The difference in active/ sham guesses between the two groups was not significant (x^2^=0.00, df =1, p=0.98).” | “raters and subjects blind to treatment group assignment.” | “Thirty-five subjects completed the five-session sham-controlled phase and 34 subjects received ten active sessions of tDCS (over both sham-controlled and open treatment phases) (see Fig. 1).”  “Intention-to-treat last observation- carried-forward scores were used for the analyses below.” | Results of all primary outcome measures were reported. |
| Blumberger,2012 | “subjects were randomly assigned using a computer generated randomization list” | “randomization list with the information stored on a centralized computer to receive either active or sham tDCS.” | “clinical raters and subjects blind to treatment group allocation. Only the treating clinician was aware of subjects’ treatment condition.”  “Of a total of 19 subjects who were assessed for maintenance of the blind, 14 subjects (73.7%) correctly guessed whether they received active or sham treatment: 6 (60.0%) in the active tDCS group and 8 (88.9%) in the sham group. These proportions did not differ significantly between the two groups (p = 0.30).” | “clinical raters and subjects blind to treatment group allocation.” | Fig. 1. CONSORT flow chart.  “Post-treatment (week 1) data on the primary outcome measure was available for n = 21 subjects (87.5%).”  “the analysis was conducted on an intention to treat basis.” | Results of all primary outcome measures were reported. |
| Brunoni, 2013 | “A research assistant not directly involved in other aspects of the trial performed a 1:1:1:1 permuted block randomization” | “the allocation was concealed using a central randomization method.” | “The raters and patients were blinded to the treatment, and contact between participants was avoided to enhance study blinding.”  “Two certified nurses administered the tDCS intervention. Because the nurses were not blinded to the intervention, their interaction with the participants was minimal. Accordingly, they did not participate in assessment of the outcomes or in any other aspect of the trial.” | “The raters and patients were blinded to the treatment, and contact between participants was avoided to enhance study blinding.” | “Nine patients dropped out within the first 2 weeks and 103 patients (85.8%) completed the entire trial (eFigure).”  “Analyses were conducted in the intention- to-treat sample according to the last observation carried forward through the time points.” | Results of all primary outcome measures were reported. |
| Brunoni, 2014 | no information | no information | Trained nurses were responsible to deliver the tDCS sessions and were instructed to adopt the same procedures for both sham and active stimulation. They were also trained to turn off the device outside patient's eyesight. | no information | “Four patients dropped-out at week 2 and 13 patients dropped-out at week 4. The dropouts were evenly distributed between groups (Fig. 1).” Applied intention-to-treat analysis.  “3 of the 40 participants were not included in our analyses due to trial abandonment (i.e., did not complete at least 2 visits to our research center, n=2) and technical reasons (n=1, one participant with bipolar depression who was mistakenly diagnosed as unipolar depression during trial enrollment). | Results of all primary outcome measures were reported. |
| Segrave, 2014 | “Using a predetermined randomization schedule constructed using a random number generator” | “Using a predetermined randomization schedule constructed using a random number generator”  “Randomization was conducted by a member of the research team with no role in outcome assessment” | “Participants were asked to guess which treatment condition they had been receiving at the end of the treatment course. Both participants and clinical raters remained blind to treatment condition until the end of the three week follow up assessment when unmasking occurred.”  “Forty four percent of those sampled (11/25) correctly guessed both the stimulation and cognitive training condition they were allocated to. Fifty six percent (14/25) correctly guessed their cognitive training condition and 68% (17/25) correctly guessed the stimulation condition they received. Differences between the groups in correct versus incorrect guess of condition allocation did not reach significance in any of these comparisons: combined stimulation and cognitive training blinding, x^2^ (2) = .26, P = .88; stimulation blinding: x^2^ (2) = 1.00, P = .61; cognitive training blinding: x^2^ (2) = .65, P = .72.” | “The primary clinical outcome measure was the MADRS, which was completed by a trained rater blind to treatment condition at baseline.”  “Both participants and clinical raters remained blind to treatment condition until the end of the three week follow up assessment when unmasking occurred.” | “One participant in the tDCS + CCT condition withdrew following completion of the full treatment course and end of treatment assessments, but prior to follow up review, due to non-response and desire to commence antidepressant medication without delay.” | Results of all primary outcome measures were reported. |
| Bennabi, 2015 | “subjects were randomly assigned using a computer-generated randomization list” | “subjects were randomly assigned using a computer-generated randomization list with the information stored on a centralized computer to receive either active or sham tDCS.” | “Predefined codes assigned to either real or sham stimulation were used to start the stimulator and thus allowed for a double-blind study design” | “A trained, licensed neuropsychologist blinded to the patients’ treatment group conducted a complete neuropsychological test battery at baseline (T1) and 30 days (T4) after the end of the treatment.” | “One patient experienced mania and was subsequently withdrawn from the trial.” | Results of all primary outcome measures were reported. |
| Loo, 2018 | “randomly assigned by a computer-generated random number sequence to active or sham tDCS with permuted-block randomization. Randomization was stratified according to whether participants were diagnosed with unipolar or bipolar depression.” | “Opaque sealed enveloped that contained codes for the assigned groups.” | “All participants, tDCS treaters, and study raters were blinded to the participants' tDCS group allocation in the RCT phase.”  “Adequacy of blinding to treatment was assessed at the end of the RCT and open label phases by asking participants and raters to guess the tDCS condition administered during the RCT phase.”  “Chi-square tests to assess blinding adequacy found no significant association between participants' (x^2^ = 0.038; p = 0.99) or observer raters' (x^2^ = 1.403; p = 0.324) guesses in the tDCS condition received and the actual tDCS condition given, with a greater percentage overall of participants and raters guessing allocation to the sham tDCS group.” | “Adequacy of blinding to treatment was assessed at the end of the RCT and open label phases by asking participants and raters to guess the tDCS condition administered during the RCT phase.”  “The rate of correct guesses for the blinded raters was 66.7% for the sham group and 44.4% for the active group.”  “Chi-square tests to assess blinding adequacy found no significant association between participants' (x^2^ = 0.038; p = 0.99) or observer raters' (x^2^ = 1.403; p = 0.324) guesses in the tDCS condition received and the actual tDCS condition given, with a greater percentage overall of participants and raters guessing allocation to the sham tDCS group.” | “A total of 60 patients were assigned to receive placebo, 91 to receive escitalopram, and 94 to receive tDCS. Of these 245 patients, 202 received all 22 planned sessions of actual or sham tDCS and completed the week-10 assessment (55 patients in the placebo group, 75 in the escitalopram group, and 72 in the tDCS group) (Fig. S1 in the Supplementary Appendix). Withdrawal rates did not differ significantly among the three groups (χ^2^=4.77, P=0.09).”  “As per the a priori study plan [13], analyses were restricted to the 120 participants with at least one postbaseline rating, and outcome measures were analysed for change over the 4-week RCT period using a mixed effects repeated measures (MERM) model with a restricted number of covariates.” | Results of all primary outcome measures reported. |
| Mayur, 2018 | “patients were randomized as per a random number list generated by a computer program to either an active tDCS or a sham tDCS” | “The random number list indicated treat with tDCS or treat with sham tDCS and was sealed in 18 separate envelops that was opened on the morning of the initial treatment with tDCS.” | no information | no information | “2 patients (1 from the ECT + tDCS and 1 from the ECT + sham) dropped out before the completion of the 6 ECT course. The dropouts were related to withdrawal of consent.” | lack of response and remission |
| Pavlova, 2018 | “The patients were randomized into three groups” “Randomization was conducted by the non-blinded researchers during the first stimulation session by pooling randomly mixed labels with the written stimulation type.” | “Randomization was conducted by the non-blinded researchers during the first stimulation session by pooling randomly mixed labels with the written stimulation type.” | “The trial was single-blinded due to the limitations of the employed tDCS equipment which did not allow the performance of double blinded trials.”  “Participants could not distinguish between real and sham stimulation (Sign Test, z = −0.5, p = 0.6).”  “caution was given to limit communication between the non-blinded researcher and patients during tDCS procedure, as well as to following the same standards (like precision of the hot spot localization) in all groups.” | “the rater of the study (clinical psychologist), trained in performance of depression scores and neuropsychological tests, was blinded to the stimulation type” | “Five patients dropped-out from the study (0/ sham, 3/20-minute, 2/30-minute): One due to change of diagnosis (20- minute group), one discharged from the hospital due to family reasons (20-minute group), two patients were discharged from the hospital due to infectious disease (30-minute group) and one because of blood pressure elevation (20-minute group).” | Results of all primary outcome measures reported. |
| Welch, 2018 | “Participants were randomly assigned to receive 12 sessions (three days per week for four weeks) of active or sham tDCS administered concurrently with 12 modules of CBT delivered via computer-based modules.” | no information | “Subjects and raters were blinded to treatment group but tDCS technicians were unblinded.”  “In regard to participant blinding, the correct condition (active or sham) was guessed by 46% of participants. Significantly more active as compared to sham participants correctly guessed their condition; five out of eight active participants guessed the correct condition while only one sham participant correctly guessed the sham condition (x^2^ = 4.064, df = 1, p < 0.05), suggesting that our sham protocol was adequate.” | “Subjects and raters were blinded to treatment group”  “The blinded condition was correctly guessed by the assessment rater in 50% of the cases.” | “Three active participants and one sham participant withdrew before completing 12 sessions because of a lack of efficacy and/or dislike of the eCBT program, and one (active) was discontinued due to onset of heart palpitations and ankle swelling that was thought to be unrelated to tDCS.” | Results of all primary outcome measures were reported. |
| Nord, 2019 | “randomization (using custom-written MATLAB code by a researcher not involved in the trial).” | “Researchers involved in the trial were given a list of five-digit codes to input to the stimulator, half of which corresponded to active stimulation and half to sham.” | “Patients, investigators, and therapists were blind to tDCS condition for the duration of the trial. 38.5% of patients correctly guessed the stimulation condition, with no significant difference in the proportion of active and sham guesses between groups (X^2^ = 1.64, p = 0.301), suggesting that blinding was effective.” | “Patients, investigators, and therapists were blind to tDCS condition for the duration of the trial.” | “Six patients did not complete the intervention (defined as ≥ 7/8 sessions: 1 active; 5 sham); dropout rates differed marginally (non-significantly) between groups (p = 0.091);”  “to account for potential non-random dropout we employed intention-to-treat analyses as our primary analysis.” | Results of all primary outcome measures were reported. |

## Figure S1. Risk of bias

1. **Ratings on risk of bias of included studies**


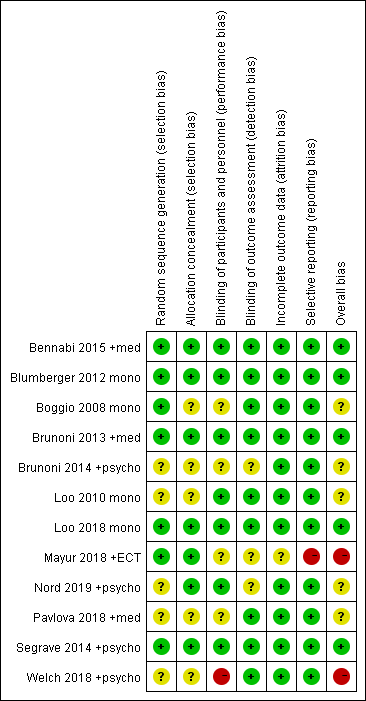


Note: mono=tDCS monotherapy; +med= tDCS + medicine; +psycho = tDCS + psychotherapy; +ECT = tDCS + ECT; green circles represent low risk; yellow circles represent unclear risk of bias; red circles represent high risk of bias.

**(b) Methodologic Characteristics of Individual Studies According to the Cochrane Risk of Bias Tool**


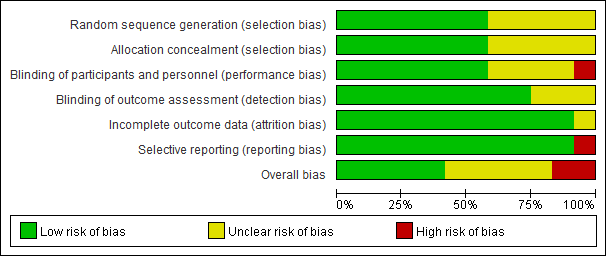

Supplement: Supplementary file 2 — Additional file 2: S3. Supporting statements. Figure S1. Risk of bias. [file 12916_2021_2181_MOESM2_ESM.docx]
