# Supplementary material for: Is transcranial direct current stimulation, alone or in combination with antidepressant medications or psychotherapies, effective in treating major depressive disorder? A systematic review and meta-analysis
Source: BMC Med. 2021 Dec 17;19:319. doi: 10.1186/s12916-021-02181-4 (PMC8680114; doi:10.1186/s12916-021-02181-4)
Supplement: Supplementary file 3 — Additional file 3: Figure S2. Cumulative meta-analysis. [file 12916_2021_2181_MOESM3_ESM.docx]

# Cumulative Meta-analysis

## Figure S2. Cumulative meta-analysis
