# Supplementary material for: Is transcranial direct current stimulation, alone or in combination with antidepressant medications or psychotherapies, effective in treating major depressive disorder? A systematic review and meta-analysis
Source: BMC Med. 2021 Dec 17;19:319. doi: 10.1186/s12916-021-02181-4 (PMC8680114; doi:10.1186/s12916-021-02181-4)
Supplement: Supplementary file 4 — Additional file 4: Table S1. Univariable meta-regression for score and acceptability. [file 12916_2021_2181_MOESM4_ESM.docx]

# Influence factors

## Table S1: Univariable meta-regression for score and acceptability

|  | **Depression score** | | | **Dropout rate** | | |
| --- | --- | --- | --- | --- | --- | --- |
|  | Coef (B) | 95% CL | P | Coef (B) | 95% CL | P |
| *Clinical characteristics* |  |  |  |  |  |  |
| Publication year | 0.058 | -0.08 to 0.19 | 0.357 | 0.156 | -0.09 to 0.40 | 0.194 |
| Baseline score ^a^  Treatment strategy | 0.025 | -0.07 to 0.12 | 0.569 | 0.012 | -0.15 to 0.17 | 0.873 |
| Monotherapy | -0.441 | -1.46 to 2.34 | 0.613 | -0.222 | -3.86 to 3.42 | 0.893 |
| tDCS +Medicine | -0.727 | -1.20 to 2.65 | 0.415 | -0.397 | -4.18 to 3.39 | 0.818 |
| tDCS +Psychotherapy | -0. 161 | -2.07 to 1.75 | 0.853 | -1.081 | -4.78 to 2.61 | 0.525 |
| *Demographics* |  |  |  |  |  |  |
| Sample size | 0.003 | 0.02 to 0.03 | 0.817 | 0.012 | -0.02 to 0.04 | 0.438 |
| Age | -0.026 | -0.10 to 0.04 | 0.430 | 0.032 | -0.10 to 0.17 | 0.612 |
| Female rate | -1.88 | -4.34 to 0.58 | 0.121 | 0.537 | -4.35 to 5.4.2 | 0.813 |
| *Montage parameters* |  |  |  |  |  |  |
| Size of electrode | 0.012 | -0.06 to 0.08 | 0.714 | -0.035 | -0.17 to 0.10 | 0.573 |
| Current intensity | 0.194 | -0.52 to 0.91 | 0.562 | 0.359 | -0.86 to 1.58 | 0.531 |
| Stimulation duration | 0.005 | -0.09 to 0.10 | 0.915 | 0.076 | -0.10 to 0.25 | 0.351 |
| Number of sessions  Total charge^b^ | 0.019  0.013 | -0.10 to 0.14  -0.03 to 0.05 | 0.729  0.494 | 0.127  0.035 | -0.03 to 0.29  -0.02 to 0.09 | 0.112  0.196 |

Note: ^a^ Baseline score was calculated by weighted arithmetic mean of depression scores of active and sham groups; ^b^ total charge = (current intensity × stimulation duration × number of sessions)/size of electrode; boldface means q<0.1.
