# Supplementary material for: Is transcranial direct current stimulation, alone or in combination with antidepressant medications or psychotherapies, effective in treating major depressive disorder? A systematic review and meta-analysis
Source: BMC Med. 2021 Dec 17;19:319. doi: 10.1186/s12916-021-02181-4 (PMC8680114; doi:10.1186/s12916-021-02181-4)
Supplement: Supplementary file 5 — Additional file 5: Figure S3. Influence analyses with random models (a) depression score by Hedges; (b) acceptability by OR; (c) response by OR; (d) remission by OR. Table S2. Results of influence analyses with random models (a) primary outcomes; (b) secondary outcomes. Figure S4. Meta-analysis (excluded high risk trials). [file 12916_2021_2181_MOESM5_ESM.docx]

# Sensitivity analysis

## Figure S3. Influence analyses with random models (a) depression score by Hedges; (b) acceptability by OR; (c) response by OR; (d) remission by OR.

1. (b)

1. (d)

## Table S2. Results of influence analyses with random models

### Primary outcomes

|  | **Depression score** | | **Dropout rate** | |
| --- | --- | --- | --- | --- |
| **Study omitted** | **Estimate** | **95% CI** | **Estimate** | **95% CI** |
| Boggio (2008) | -0.241 | -0.547, 0.065 | 0.580 | 0.281, 1.196 |
| Loo (2010) | -0.422 | -0.818, -0.026 | 0.649 | 0.303, 1.391 |
| Blumberger (2012) | -0.414 | -0.806, -0.021 | 0.604 | 0.288, 1.268 |
| Brunoni (2013) | -0.343 | -0.746, 0.059 | 0.647 | 0.301, 1.389 |
| Brunoni (2014) | -0.408 | -0.809, -0.007 | 0.634 | 0.297, 1.355 |
| Segrave (2014) | -0.416 | -0.804, -0.029 | 0.580 | 0.281, 1.196 |
| Bennabi (2015) | -0.296 | -0.661, 0.069 | 0.599 | 0.285, 1.257 |
| Loo (2018) | -0.432 | -0.826, -0.038 | 0.415 | 0.182, 0.947 |
| Mayur (2018) | -0.423 | -0.806, -0.041 | 0.560 | 0.266, 1.181 |
| Pavlova (2018)^1^ | -0.300 | -0.670, 0.070 | 0.540 | 0.257, 1.136 |
| Pavlova (2018)^2^ | -0.362 | -0.760, 0.037 | 0.519 | 0.247, 1.093 |
| Welch (2018) | -0.391 | -0.775, -0.006 | 0.563 | 0.263, 1.209 |
| Nord (2019) | -0.385 | -0.792, 0.022 | 0.679 | 0.316, 1.457 |
| **Combined** | -0.371 | -0.740, -0.002 | 0.580 | 0.281, 1.196 |

Note: ^1^ the stimulation duration of active group is 30 minutes; ^2^ the stimulation duration of active group is 20 minutes

### Secondary outcomes

|  | **Response rate** | | **Remission rate** | |
| --- | --- | --- | --- | --- |
| **Study omitted** | **Estimate** | **95% CI** | **Estimate** | **95% CI** |
| Boggio (2008) | 1.475 | 0.914, 2.381 | 1.176 | 0.615, 2.248 |
| Loo (2010) | 1.540 | 0.940, 2.524 | 1.210 | 0.585, 2.503 |
| Blumberger (2012) | 1.549 | 0.967, 2.482 | 1.265 | 0.669, 2.395 |
| Brunoni (2013) | 1.394 | 0.847, 2.297 | 1.242 | 0.586, 2.633 |
| Brunoni (2014) | 1.577 | 0.968, 2.568 | 1.359 | 0.699, 2.643 |
| Segrave (2014) | 1.606 | 1.014, 2.544 | 1.272 | 0.630, 2.567 |
| Bennabi (2015) | 1.501 | 0.923, 2.439 | 1.214 | 0.608, 2.426 |
| Loo (2018) | 1.865 | 1.151, 3.023 | 1.552 | 0.854, 2.820 |
| Pavlova (2018) ^1^ | 1.353 | 0.852, 2.150 | 1.082 | 0.561, 2.085 |
| Pavlova (2018) ^2^ | 1.477 | 0.907, 2.404 | 1.311 | 0.639, 2.690 |
| Welch (2018) | 1.566 | 0.992, 2.472 | 1.369 | 0.733, 2.558 |
| Nord (2019) | 1.456 | 0.890, 2.383 | 1.117 | 0.575, 2.171 |
| **Combined** | 1.525 | 0.975, 2.385 | 1.265 | 0.669, 2.395 |

Note: ^1^ the stimulation duration of active group is 30 minutes; ^2^ the stimulation duration of active group is 20 minutes

## Figure S4. Meta-analysis (excluded high risk trials)
