# Supplementary material for: Is transcranial direct current stimulation, alone or in combination with antidepressant medications or psychotherapies, effective in treating major depressive disorder? A systematic review and meta-analysis
Source: BMC Med. 2021 Dec 17;19:319. doi: 10.1186/s12916-021-02181-4 (PMC8680114; doi:10.1186/s12916-021-02181-4)
Supplement: Supplementary file 6 — Additional file 6: Figure S5. Funnel plots by treatment strategy (a) depression score; (b) acceptability; (c) response rate; (d) remission rate. Table S3. Egger’s test for each outcome. [file 12916_2021_2181_MOESM6_ESM.docx]

# Publication bias

## Figure S5: Funnel plots by treatment strategy (a) depression score; (b) acceptability; (c) response rate; (d) remission rate

1. Depression score (b) Acceptability

1. Response rate (d) Remission rate

## Table S3. Egger's test for each outcome

| Treatment strategy | **Depression score** | | **Acceptability** | | **Response rate** | | **Remission rate** | |
| --- | --- | --- | --- | --- | --- | --- | --- | --- |
|  | Egger's | p | Egger's | p | Egger's | p | Egger's | p |
| monotherapy | -5.75 | 0.291 | -1.44 | 0.339 | 1.36 | 0.537 | 0.98 | 0.889 |
| +medicine | -3.11 | 0.321 | 2.86 | 0.331 | 2.34 | 0.613 | 0.34 | 0.889 |
| +psychotherapy | 0.84 | 0.481 | 1.31 | 0.536 | -1.91 | 0.152 | -4.04 | 0.037 |
| **Overall ^a^** | -1.53 | 0.155 | -0.35 | 0.734 | -0.34 | 0.742 | -1.00 | 0.342 |

^a^ tests for publication bias without classifying into different treatment strategy.
