# Supplementary material for: Is transcranial direct current stimulation, alone or in combination with antidepressant medications or psychotherapies, effective in treating major depressive disorder? A systematic review and meta-analysis
Source: BMC Med. 2021 Dec 17;19:319. doi: 10.1186/s12916-021-02181-4 (PMC8680114; doi:10.1186/s12916-021-02181-4)
Supplement: Supplementary file 7 — Additional file 7: Table S4. study characteristics for each trial with tDCS + medication therapy. S4. The list of included studies: tDCS + medication therapy. S5. Further investigation of tDCS + medication therapy. Figure S6. The influence of therapies of tDCS plus SSRIs only or SSRIs with other medications on unipolar depression or bipolar disorder. [file 12916_2021_2181_MOESM7_ESM.docx]

# Further investigation of tDCS + medication therapy

## Table S4. study characteristics for each trial with tDCS + medication therapy

| **Study** | **Study design** | **N** ^4^ | **Medicines** | **Target areas** | **Parameters** | **Efficacy** |
| --- | --- | --- | --- | --- | --- | --- |
| Ferrucci et al., 2009 ^a b^ | Open-label: single active group | 14 | TCA, MAOIs, SSRIs, SNRIs, NaSSA, BDZ | Anodal: left DLPFC;  Cathodal: right DLPFC | 2 mA for 20 minutes, twice a day, 5 weekdays | Positive |
| Brunoni et al., 2011 ^b^ | Open-label: single active group | 31 | TCA, SSRIs, SNRIs, NaSSA, NDRI, LMT, DVP, QTP, OLZ, ARP, Li | Anodal: left DLPFC;  Cathodal: right DLPFC | 2 mA for 20 minutes, twice a day, 5 weekdays | Positive |
| Dell’Osso et al., 2012 ^a b^ | Open-label: single active group | 23 | SSRIs, SNRIs or TCAs | Anodal: left DLPFC; Cathode: contralateral cortical area | 2 mA for 20 minutes, twice a day, 5 weekdays | Positive |
| Palm et al., 2012 ^a^ | Cross-over study^3^ | 11 | TCA, MAOIs, SSRIs, SNRIs, combinations or added antipsychotics, mood stabilizers, or anxiolytics | Anode: left DLPFC;  Cathode: contralateral supraorbital region | 1 or 2 mA for 20 minutes per day, 10 weekdays | Not superior |
| Brunoni et al., 2013 ^1^ | RCT | 30 | Sertraline hydrochloride, 50 mg/d | Anodal: left DLPFC;  Cathodal: right DLPFC | 2 mA for 30 minutes per day, 10 weekdays | Positive |
| Brunoni et al., 2013 ^a b 2^ | Naturalistic study: single active group | 82 | SSRIs, SNRIs, antipsychotics, 5HT, dopaminergic receptor | Anodal: left DLPFC;  Cathodal: right DLPFC | 2 mA for 20 minutes, twice a day, 5 weekdays | Positive: TCAs, dual-reuptake inhibitors;  Negative: BDZ;  Not superior: SSRIs; |
| Chan et al., 2013 ^a b^ | Open-label:  single active group | 5 | D-Cycloserine, 100-mg | Anode: left DLPFC;  Cathode: F8 | 2 mA for 20 minutes per day, 20 weekdays | Not superior |
| Dell’Osso et al., 2014 ^a^ | Open, follow-up study: single active group | 23 | Mixed medicines: half for SSRIs, the addition of a BDZ for a part of subjects | Anode: left DLPFC;  Cathode: contralateral supraorbital region. | 1 or 2 mA for 20 minutes per day, 10 weekdays | Inconclusive: sustained effects but significant more dropouts. |
| Bennabi et al., 2015 | RCT | 23 | Escitalopram, 10-20 mg/day | Anode: left DLPFC;  Cathode: contralateral supraorbital area | 2 mA for 30 minutes, twice a day, 5 weekdays | Positive |
| Pavlova et al., 2018 | RCT | 48 | Sertraline, 50 mg/day | Anodal: left DLPFC;  Reference: contralateral orbit | 0.5 mA for 20/30 minutes, 10 weekdays | Positive |
| Li et al.,  2019 ^a b^ | Single active group | 18 | 16/18 with antidepressants;  17/18 with BDZ; | Anodal: left DLPFC;  Cathodal: right DLPFC | 2 mA for 30 minutes per day, 10 weekdays | Positive |
| Palm et al., 2019 ^b^ | Single active group | 24 | Escitalopram/citalopram (concomitant: hydroxyzine, antipsychotics, BDZ) | Anodal: left DLPFC;  Cathodal: F4 | 2 mA for 30 minutes, twice a day, 10 weekdays | Positive |
| Zhou et al., 2020 ^c^ | RCT | 47 | Escitalopram and Zopiclone | Anodal: left DLPFC;  Cathodal: right DLPFC | 2 mA for 30 minutes per day, 20 weekdays | Positive |

Note: Exclusion reasons in meta-analysis: ^a^ included bipolar patients and cannot be separated from the sample; ^b^ lack of sham-control group; ^c^ lack of main outcomes; ^d^ cannot exclude the placebo effect; ^e^ duplicative dataset from the same trial. ^1^ this study is Brunoni et al. (2013a) listed in S3; ^2^ this study is Brunoni et al. (2013b) listed in S3; ^3^ this is a cross-over study, we only utilized the data from the first phase; ^4^ N = the number of tDCS + medicine group. Antidepressants: SSRI (Selective Serotonin Reuptake Inhibitors), SNRI (Serotonin–Noradrenalin Reuptake Inhibitors), TCA (Tricyclic Antidepressants), NaSSA (Noradrenergic and Specific Serotonergic Antidepressants), MAOI (Monoamine Oxidase Inhibitor); NDRI (Noradrenaline and Dopamine Reuptake Inhibitors); LMT (lamotrigine); DVP (sodium divalproate); QTP (quetiapine); OLZ (olanzapine); ARP (aripiprazole); Li (Lithium); VNF (Venlafaxine ER); BDZ (Benzodiazepines).

## S4. The list of included studies: tDCS + medication therapy

1. Ferrucci, R., Bortolomasi, M., Vergari, M., Tadini, L., Salvoro, B., Giacopuzzi, M., . . . Priori, A. (2009). Transcranial direct current stimulation in severe, drug-resistant major depression. Journal of Affective Disorders, 118(1), 215-219. http://www.sciencedirect.com/science/article/pii/S0165032709000822.
2. Brunoni, A. R., Ferrucci, R., Bortolomasi, M., Vergari, M., Tadini, L., Boggio, P. S., . . . Priori, A. (2011). Transcranial direct current stimulation (tDCS) in unipolar vs. bipolar depressive disorder. Progress in Neuro-Psychopharmacology and Biological Psychiatry, 35(1), 96-101. <http://www.sciencedirect.com/science/article/pii/S0278584610003611>.
3. Dell’Osso, B., Zanoni, S., Ferrucci, R., Vergari, M., Castellano, F., D’Urso, N., . . . Altamura, A. C. (2012). Transcranial direct current stimulation for the outpatient treatment of poor-responder depressed patients. European Psychiatry, 27(7), 513-517. <http://www.sciencedirect.com/science/article/pii/S0924933811000332>.
4. Palm, U., Schiller, C., Fintescu, Z., Obermeier, M., Keeser, D., Reisinger, E., Pogarell, O., Nitsche, M. A., Möller, H.-J., & Padberg, F. (2012). Transcranial direct current stimulation in treatment resistant depression: A randomized double-blind, placebo-controlled study. Brain Stimulation, 5(3), 242–251. <https://doi.org/10.1016/j.brs.2011.08.005>
5. Brunoni, A.R., Ferrucci, R., Bortolomasi, M., Scelzo, E., Boggio, P.S., Fregni, F., . . ., & Priori, A. (2013 a). Interactions between transcranial direct current stimulation (tDCS) and pharmacological interventions in the Major Depressive Episode: Findings from a naturalistic study. European Psychiatry, 28(6), 356-361. <https://doi.org/10.1016/j.eurpsy.2012.09.001>
6. Brunoni, A. R., Valiengo, L., Baccaro, A., Zanao, T. A., de Oliveira, J. F., Goulart, A., . . . Fregni, F. (2013 b). The sertraline vs. electrical current therapy for treating depression clinical study: results from a factorial, randomized, controlled trial. JAMA Psychiatry, 70(4), 383-391. doi:10.1001/2013.jamapsychiatry.32
7. Chan, H. N., Alonzo, A., Martin, D. M., Mitchell, P. B., Sachdev, P., & Loo, C. K. (2013). Augmenting transcranial direct current stimulation with (D)-cycloserine for depression: a pilot study. J ect, 29(3), 196-200. doi:10.1097/YCT.0b013e3182801b09
8. Dell'Osso, B., Dobrea, C., Arici, C., Benatti, B., Ferrucci, R., Vergari, M., . . . Altamura, A. C. (2014). Augmentative transcranial direct current stimulation (tDCS) in poor responder depressed patients: a follow-up study. CNS Spectr, 19(4), 347-354. doi:10.1017/s1092852913000497
9. Bennabi, D., Nicolier, M., Monnin, J., Tio, G., Pazart, L., Vandel, P., & Haffen, E. (2015). Pilot study of feasibility of the effect of treatment with tDCS in patients suffering from treatment-resistant depression treated with escitalopram. Clin Neurophysiol, 126(6), 1185-1189. doi:10.1016/j.clinph.2014.09.026
10. Pavlova, E. L., Menshikova, A. A., Semenov, R. V., Bocharnikova, E. N., Gotovtseva, G. N., Druzhkova, T. A., . . . Guekht, A. B. (2018). Transcranial direct current stimulation of 20- and 30-minutes combined with sertraline for the treatment of depression. Prog Neuropsychopharmacol Biol Psychiatry, 82, 31-38. doi:10.1016/j.pnpbp.2017.12.004
11. Li, M.-S., Du, X.-D., Chu, H.-C., Liao, Y.-Y., Pan, W., Li, Z., & Hung, G. C.-L. (2019). Delayed effect of bifrontal transcranial direct current stimulation in patients with treatment-resistant depression: a pilot study. BMC Psychiatry, 19(1). doi:10.1186/s12888-019-2119-2
12. Palm U, Goerigk S, Kirsch B, Bäumler L, Sarubin N, Hasan A, Brunoni AR, Padberg F. Treatment of major depression with a two-step tDCS protocol add-on to SSRI: Results from a naturalistic study. Brain Stimul. 2019 Jan-Feb;12(1):195-197. doi: 10.1016/j.brs.2018.10.003. Epub 2018 Oct 3. PMID: 30314900.
13. Zhou, Q., Yu, C., Yu, H., Zhang, Y., Liu, Z., Hu, Z., … Zhou, D. (2020). The Effects of Repeated Transcranial Direct Current Stimulation on Sleep Quality and Depression symptoms in Patients with Major Depression and Insomnia. Sleep Medicine. doi:10.1016/j.sleep.2020.02.003

## S5. Further investigation of tDCS + medication therapy

To confirm the efficacy of tDCS + medication therapy and compare the effects among different medicine combinations, we further investigated the experimental outcomes of all trials applied for tDCS + medicine by lowering our inclusion criteria to include more trials. We found ten more trials from the excluded literature list (additional file S1, and see study characteristics in Table S1), without limiting their study design, disease type, and outcome measures. Thirteen trials (additional file S4) were included in the final. Improvement and response rate were used to reflect trials’ efficacies because only they had enough numbers to compare. As shown in Table S4, most trials (10/13) represented positive outcomes, but their outcome measures varied. Rather than trials with mixed medications, trials only with SSRIs show consistent positive effects, greater improvement (mean = 50.5%), and response rate (mean = 58.8%). The result of Mann-Whitney U-test presented that the improvement of the SSRIs group (SSRIs only) was superior to it in the mix group (SSRIs with other medications) (mean = 19.4%) (p = 0.007), and the response rate of the SSRIs group was significantly higher than the mix-group’ s (mean = 26.3%) (p = 0.042).

Additionally, population is another influence factor effects the efficacy of tDCS + medication therapy. The result showed that the improvement of the unipolar group (mean = 44.9%) was superior to it in the mix (unipolar + bipolar) group (mean = 19.1%) (p = 0.018), although the response rate of the unipolar group (mean = 49.4%) was not significantly different from the mix-group’ s (mean = 28.4%) (p = 0.27).

## Fig. S6 The influence of therapies of tDCS plus SSRIs only or SSRIs with other medications on unipolar depression or bipolar disorder.


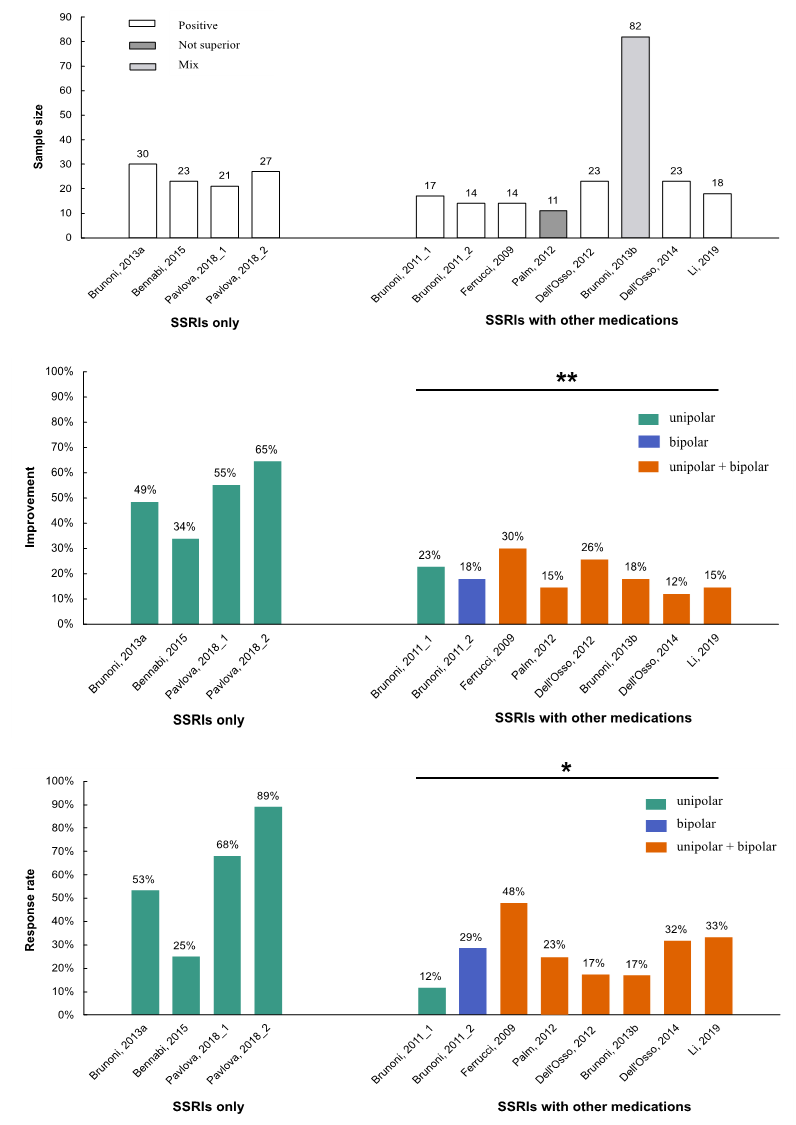


**Fig. S6** The influence of therapies of tDCS plus SSRIs only or SSRIs with other medications on unipolar depression or bipolar disorder. (a) Both therapies of tDCS + SSRIs only and tDCS + SSRIs with other medications showed positive efficacy with only one not superior to sham. (b) The therapy of tDCS + SSRIs was mainly used for unipolar depression and showed significantly greater improvement than the therapy of tDCS + SSRIs with other medications. (c) The response rate of tDCS + SSRIs therapy was significantly higher than the therapy of tDCS + SSRIs with other medication

*Note*: ** p<0.01, * p<0.05 vs. SSRIs only group, Wilcoxon rank-sum test; the horizontal line represents a comparison of two groups; the bracket represents regarding the trials below as one group. Pavlova, 2018_1, the group with 20 minutes stimulation; Pavlova, 2018_2, the group with 30 minutes stimulation; Trials Pavlova et al. (2018) and Brunoni et al. (2011) were separated into two independent data because they designed two tDCS + medication combinational groups. All percent values refer to the results of the active groups. Improvement is defined as the rate of the reduced score and initial score. Response rate is defined as >50% improvement in HDRS/MADRS.
